# Whole-Cell Glycerol-free Fluorescence Cryo-Spectroscopy Reveals Electronic Excitation Dynamics in the Diatom *Cyclotella meneghiniana*

**DOI:** 10.1021/jacsau.6c00630

**Published:** 2026-07-08

**Authors:** Lena Golubewa, Yaraslau Padrez, Patricija Zemaityte, Danielis Rutkauskas, Claudia Büchel, Andrius Gelzinis

**Affiliations:** † Institute of Chemical Physics, Faculty of Physics, 54694 Vilnius University, Sauletekio Av. 9, III bld. 10222, Vilnius, Lithuania; ‡ Department of Molecular Compound Physics, 226274 State Research Institute Center for Physical Sciences and Technology, Sauletekio Av. 3 10257, Vilnius, Lithuania; § Neuroscience Research Center, College of Medicine, 66559 Jiaxing University, Jiaxing, Zhejiang Province 314001, China; ∥ Life Science Center, Vilnius University, Sauletekio Av. 7 10257, Vilnius, Lithuania; ⊥ Institute of Molecular Biosciences, Department of Biosciences, Goethe University Frankfurt, Max-von-Laue-Str. 9 60438, Frankfurt, Germany

**Keywords:** cryo-spectroscopy, whole-cell, Cyclotella meneghiniana, diatoms, electronic excitation dynamics, glycerol, intact-frozen state, photosynthesis

## Abstract

Diatoms possess a unique photosynthetic apparatus that adapts to constant, extreme changes in light intensity, enabling efficient light harvesting and photoprotection. Fluorescence spectroscopy studies at cryogenic temperatures can reveal details of the electronic excitation dynamics in these evolutionarily successful organisms. Whole-cell studies are highly valuable since they allow investigation of the photosynthetic machinery rather than separate complexes affected by isolation. In this study, we propose an approach that enables cryogenic fluorescence analysis of photosynthetic processes in the diatom *Cyclotella meneghiniana*, frozen in an intact state at 16–130 K. We show the drastic effects on cell viability caused by traditionally used glycerol-containing cryo-media, including rapid cellular damage (with about 36% of cells losing membrane integrity immediately upon medium exchange, reaching >99% within 3.5 h), thylakoid membrane disruption, likely detachment of fucoxanthin chlorophyll proteins (FCPs) from photosystem II (PSII) cores, and possible FCP aggregation. By omitting glycerol, we provide whole-cell cryo-measurements of the photosynthetic apparatus in cells frozen in an intact state and demonstrate the retention of some function (e.g., activation of alternative electron transfer) of the photosynthetic complexes in damaged cells, which, however, differ from those in intact cells. We identified specific features of intact whole-cell temperature-dependent excitation dynamics, including a gradual change in the relative contributions of CP47 (690 nm) and CP43 (685 nm) emissions, slowed charge separation and energy transfer within PSII cores at low temperatures, and a monotonic increase in PSI-FCP emission at 700 nm with decreasing temperature. Our results indicate that cryo-spectroscopy data obtained in glycerol-containing media should be interpreted with caution: cell viability must be ensured before measurements, and pseudofunctioning of the photosynthetic machinery in damaged cells should be considered. This approach provides a reliable framework for investigating intact photosynthetic cells and offers new insights into the complexity of excitation dynamics.

## Graphical abstract

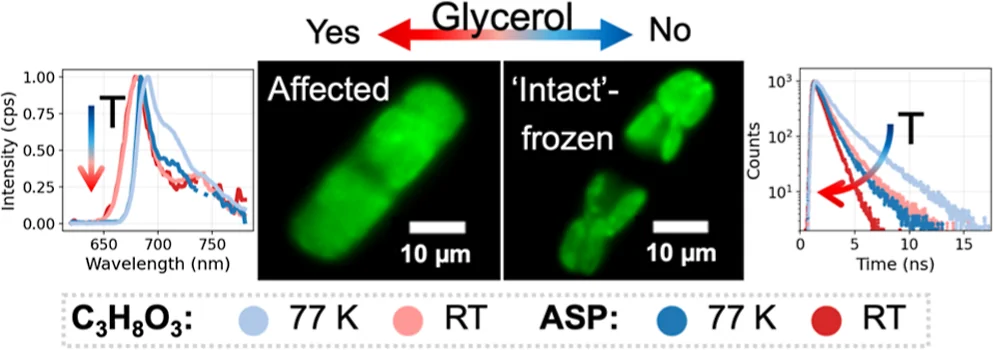

## 1 Introduction

Diatoms are unicellular algae found in nearly all aquatic habitats^1^ and account for almost a quarter of global primary production.^2–4^ They play a key role in major biochemical cycles on Earth, contributing significantly to the cycles of carbon, silicon, nitrogen, and phosphorus.^5^

Diatoms possess unique systems for light harvesting and very efficient photoprotective mechanisms and exhibit rapid adaptation to changing environments, which determine their evolutionary success.^6^ A mechanistic understanding of these features is highly important for both fundamental and applied sciences.^7^

The centric diatom *C. meneghiniana* is one of the most actively studied diatoms to date. Advances in techniques for purifying the fucoxanthin (Fx)-chlorophyll (Chl) *a*/*c*-binding protein (FCP) light-harvesting complexes from this diatom^8^ and the precise elucidation of their protein composition^9^ have accelerated the analysis of their internal energy transfer dynamics. However, despite the discovery of the structures of FCP antennae and photosystem II (PSII)-FCP supercomplex^10^ as well as the presence of ultrafast spectroscopic experiments providing the benchmarks for the analysis of the structural data,^11^ the understanding of the photosynthetic machinery and features of energy transfer dynamics in these organisms remains incomplete.

Studies of electronic excitation dynamics are mainly performed on isolated structures, including FCPs or PS-FCP supercomplexes, which provide valuable information but are limited, to some extent. A lot of studies of intermolecular excitation transfer are conducted on solubilized FCPs.^12,13^ Studies of FCP aggregates additionally provide information on intercomplex interactions and energy transfer.^14–16^ Incorporation of FCP antenna complexes into liposomes^17^ broadens the spectrum of processes available for study, as it more closely resembles the cellular environment.^18^

Despite the evident progress reached on isolated structures, experimental limitations restrict understanding of the interplay between PSs and FCPs as well as their crosstalk with other cellular compartments. Isolation and purification procedures have drastic effects on components of the photosynthetic apparatus, casting doubt on the possibility of extrapolating conclusions obtained from isolated complexes to the organismal level. Studies of intermolecular/intercomplex excitation energy transfer and photoprotection at the level of a whole cell, preserving its integrity and viability, are therefore of the highest importance.^19–21^

Fluorescence spectroscopy enables the revealing of the molecular aspects of the functioning of the photosynthetic apparatus of diatoms. A combination of steady-state and time-resolved fluorescence spectroscopy at cryogenic temperatures is a powerful tool for studying dynamic photosynthetic processes. Cooling to cryogenic temperatures enhances spectral resolution due to band narrowing and enables observation of specific processes by “trapping” molecular systems in specific conformational states (e.g., quenched, relaxed, and recovered states^21^) that may be unstable at room temperature (RT) or reveal the complex interplay between FCPs and PS cores.^22^ Such cryogenic measurements provide a new understanding of a functioning photosynthetic apparatus at the whole-cell level. Unfortunately, the number of such studies is rather limited to this day.

Traditionally, cryogenic measurements of cells are performed in a mixture of medium (such as artificial seawater, ASP) and an additive intended to suppress light scattering caused by ice crystal formation. In protein studies, sugars (trehalose and sucrose), betaine, or glycerol are often used.^23–25^ Glycerol concentrations of 20–30% are often used in studies of protein structure by cryoelectron microscopy (cryo-EM), where glycerol preserves proteins from undesired disassembly or aggregation.^26^ However, in low-temperature spectroscopy experiments on algae or cyanobacteria cells, plastids, or thylakoid membranes, glycerol is commonly^27–30^ but not universally^31–33^ added to growth medium (e.g., ASP), at a 1:1 or 2:3 ratio,^34–36^ to prevent phase separation and water crystallization, thereby maintaining sample transparency, which is fully achieved only at a 65% w/w (60% v/v) fraction of glycerol.^34,37^ In most studies, it is assumed that the cells transferred to such media remain intact, and the similarity of spectral shapes is taken as evidence for this assumption.^38^ However, no additional cell viability tests are often provided,^21,22,38,39^ although cell exposure to such nonphysiological mixtures can lead to significant changes in cell condition and thylakoid functionality, as noticed for cyanobacteria^40,41^ and for *Phaeodactylum tricornutum*.^27,42^ Consequently, the entire photosynthetic machinery of the cell may be disrupted, leading to inconsistent results and interpretive errors.

In this study, we conducted steady-state and time-resolved fluorescence analyses of intact cells of the diatom *C. meneghiniana* at temperatures ranging from 16 K to RT to reveal the interplay of FCPs and PSs at the whole-cell level. To achieve this, we first verified cell integrity and viability before cryogenic measurements. We identified time-dependent changes in the spectroscopic properties of *C. meneghiniana* cells after transferring them to an ASP/glycerol (2:3 ratio) mixture prior to cryofluorescence measurements and showed that traditional cell viability estimation, which is based on comparing the shapes and maximum positions of the fluorescence spectra before and after media exchange, does not reflect the actual state of the cells and cannot verify the condition of the photosynthetic apparatus. We demonstrated a decrease in cell viability in ASP/glycerol and observed morphological changes accompanied by significant alterations in far-red fluorescence at cryogenic temperatures, reflecting disassembly of the photosynthetic apparatus, possible detachment of FCPs from PS cores, and FCP aggregation. By avoiding glycerol, we maintained cell integrity during cryogenic measurements and provided an analysis of electronic excitation dynamics at the whole-cell level at temperatures from 16 to 130 K and RT. In addition to changes in the relative intensities of CP43 and CP47 emissions and slower fluorescence decays at 685 nm, which reflect slower charge separation and energy transfer to the fluorescing species in the PSII cores at low temperatures, cryo-spectroscopy measurements revealed a monotonic increase in the broad red emission above 700 nm with decreasing temperature, which is more likely dominated by PSI-FCP complexes, with no clear contributions from the FCP complexes forming the PSII antenna. Importantly, these signatures differ from those observed in glycerol-affected cells or isolated complexes, demonstrating that native-state cryospectroscopy captures the interconnected photosynthetic chain rather than its disconnected components.

## 2 Materials and Methods

### 2.1 *Cyclotella meneghiniana* Cells

*C. meneghiniana* cells (strain 1020-1a, Göttingen) were cultivated at low light (20 μmol photons/(m^2^ s)) in a 16:8 h light/dark regime at 15 °C with continuous stirring in ASP^43^ supplemented with 2 mM silica. After 1 week (midexponential growth, light-adapted state), cells were harvested and prepared for measurements as follows: concentration was adjusted based on the optical density (OD) at the Chl *a* Q_*y*_ band (the S_1_–S_0_ transition of Chl *a*), the initial UV–vis absorbance spectrum was measured, cells were gentle centrifuged (500 rcf, 8 min, RT) and resuspended in ASP or a medium/glycerol (2:3 ratio) to a final OD of 0.05–0.10 (Chl *a* Q_*y*_ band), followed by 10–15 min dark adaptation prior to measurement.

### 2.2 UV–Vis Absorbance Measurements

Ultraviolet–visible (UV–vis) absorbance spectra of *C. meneghiniana* cell suspensions were measured in a 1 cm path length quartz cuvette using a JASCO V-670 UV–vis spectrophotometer (Jasco, USA).

### 2.3 Steady-State and Time-Resolved Fluorescence Measurements

Steady-state fluorescence emission spectra and time-resolved fluorescence decay measurements were performed using a time-correlated single-photon-counting fluorescence spectrometer FLS920 (Edinburgh Instruments Ltd., UK). A semiconductor diode laser EPL-375 (Edinburgh Instruments Ltd., UK), λ_*ex*_ = 371 nm, repetition rate of 5 MHz, and an energy of 1 nJ/pulse, was used throughout the studies. The excitation wavelength used enabled direct excitation of mainly Chl *a* and Chl *c*, minimizing excitation via fucoxanthins and thus providing information on excitation dynamics in both light-harvesting antennae and PSI/PSII cores. Spectra were collected in the 600–780 nm range with an integration of 1 s. Fluorescence decays were measured at emission wavelengths of 685 and 720 nm with an emission slit of 20 nm in a 20 ns window. The full width at half-maximum (FWHM) of the instrument response function (IRF) was 345 ps.

RT measurements were performed in a fused silica cell with a 1 cm optical-path length. Measurements at cryogenic temperatures were performed with a closed-cycle helium cryostat (Janis Research Company, LLC) by using a fused silica cell with a 0.5 mm optical path length. The OD values of the cell suspensions were adjusted to between 0.05 and 0.1. All fluorescence measurements were performed in a 90° detection geometry.

After 10 min of dark adaptation, the *C. meneghiniana* cell suspension in a fused silica cell was placed in the cryostat. Steady-state fluorescence spectra and time-resolved fluorescence decays at 685 and 720 nm were measured at RT. The temperature was then lowered to 16 K, and measurements were repeated during stepwise warming to RT. Afterward, the thawed cell sample was left in a cryostat overnight. Twelve hours later (approximately 24 h after the start), the sample was removed, mixed to redistribute the sediment evenly, and measured again. The workflow is demonstrated in Figure S1.

Measurements at cryogenic temperatures were performed on untreated control cells or on cells treated with either DCMU (3-(3,4-dichlorophenyl)-1,1-dimethylurea, an inhibitor of PSII) or FCCP (carbonyl cyanide *p*-trifluoromethoxyphenylhydrazone, a proton ionophore). In the case of the inhibitor-treated cells, the concentrations of DCMU and FCCP were selected based on dose–response measurements (Figure S2) before the measurements at cryogenic temperatures. The concentrations 20 μM DCMU and 250 nM FCCP were finally chosen for the cryogenic temperature measurements since (i) no significant differences were revealed between the screened DCMU concentrations and 20 μM DCMU was selected as one of the most commonly used concentration^44–48^ to ensure better comparison of the results with other published data; (ii) obvious concentration dependence of spectral features was revealed for FCCP and the intermediate concentration was selected to avoid FCCP-caused damage and at the same time to enable detectable effects on the spectral features of the cells (Figure S3).

Measurements at cryogenic temperatures were performed at least 3–5 times. In each measurement, the cells were exposed to all temperatures, and by the end of the measurement, the cells were damaged. Therefore, each subsequent measurement was performed on fresh cells from different cultures.

### 2.4 *Cyclotella meneghiniana* Cell Viability Analysis with Propidium Iodide

Membrane integrity of *C. meneghiniana* cells exposed to different media was tested according to ref 49 using the fluorescent dye propidium iodide (PI). Briefly, cells were incubated with 10^−6^ M PI (Sigma-Aldrich) for 30 min in the dark and then exposed to different media (also containing PI). PI is nonfluorescent until it intercalates between the bases of DNA molecules. If membrane integrity is intact, PI does not enter the cell, and no fluorescence is observed (PI: λ_*ex*_ = 535 nm, λ_em_^max^ = 615 nm). When membrane integrity is compromised, PI binds nucleic acids by intercalating between the bases and becomes strongly fluorescent.

PI spectra in samples were measured in 1 cm quartz cuvettes using a Fluorometer FS5 (Edinburgh Instruments Ltd., UK). Samples of cells in different media were prepared with and without PI. The integrated fluorescence intensity (550–650 nm) of the samples without PI was subtracted from the integrated intensity of the cell samples with PI and normalized by the integrated intensity difference between the positive and negative controls (formula 1 and 2). A suspension of cells in ASP kept for 15 min at 90 °C was used as a positive control, and the corresponding integrated intensity was considered as 100% dead cells, while a suspension of cells in ASP at RT without any treatment was considered as 0% dead cells (all cells viable).

1 $$\left[Portion\;of\;dead\;cells\right]=\frac{I_{i}^{PI}-I_{i}}{I_{\Sigma }^{+}-I_{\Sigma }^{-}}\cdot 100\%$$

where

2 $$I_{\Sigma }^{+}=I_{+}^{PI}-I_{+},I_{\Sigma }^{-}=I_{-}^{PI}-I_{-}$$

and *I*_*i*_^PI^ is the integrated fluorescence intensity of the *i*-th sample stained with PI in the spectral range [550; 650] nm, *I*_*i*_ is the integrated fluorescence intensity of the *i*-th sample without PI in the same spectral range, “+” indicates the positive control sample, and “-” corresponds to the negative control sample.

### 2.5 Fluorescence Microscopy

For fluorescence microscopy, the cell suspension was drop-cast onto Cell-Tak-treated glass slides (Corning) and coverslip-mounted. After 10 min dark adaptation, morphological changes in the organelles within the cells were identified using wide-field fluorescence microscopy with 405 nm diode laser excitation. At least ten 200 μm^2^ areas per sample were imaged, with approximately 50 to 60 cells analyzed per sample.

Raw image stacks (20 frames/field) were preprocessed by using a stability-based fusion algorithm. Images were normalized to a uniform intensity scale, after which a stability mask was generated based on pixel consistency across frames (similarity threshold of 0.7). Stable regions were selectively averaged to reduce noise, while volatile areas retained original values from the first frame to preserve the structural details.

### 2.6 Fluorescence Decay Fitting and Data Analysis

Time-correlated single-photon counting (TCSPC) data sets were analyzed using the LifeFit Python library,^50,51^ which follows a common reconvolution technique for TCSPC data. It combines the use of trust region reflective optimization of the exponential lifetimes and IRF shift together with a non-negative least-squares (NNLS) solver for the amplitudes. A detailed algorithm description is provided in the Supporting Information.

Fluorescence decays were fitted with two- or three-exponential functions, depending on the fitting quality. The number of exponentials was selected based on the χ^2^ value (fit was considered acceptable if χ^2^ ≤ 1.5). Additionally, weighted residuals plots were analyzed (weighted residuals were centered around zero; no clear waves or trends were observed; approximately 95% of points are within ±2). An example of the 1-, 2-, 3-, and 4-exponential fits, corresponding χ^2^, and weighted residual plots are shown in Figure S17. Characteristic lifetimes τ_*i*_, intensity average lifetimes τ_AVG_, pre-exponential factors α_*i*_ (amplitudes), and fractional contributions *f*_*i*_ were extracted for further analysis. The intensity average lifetimes were calculated as follows

3 $$\tau_{AVG}=\frac{\sum_{i=1}^{n}\alpha_{i}\tau_{i}^{2}}{\sum_{i=1}^{n}\alpha_{i}\tau_{i}}$$

and fractional contributions were calculated according to

4 $$f_{i}=\frac{\alpha_{i}\tau_{i}}{\sum_{j=1}^{n}\alpha_{j}\tau_{j}}$$

where τ_*i*_ is the fluorescence lifetime of the *i*-th decay component and α_*i*_ is the amplitude (pre-exponential factor) of the *i*-th component.

Due to excitation with λ_ex_ = 371 nm and high scattering by the sample, a sharp 2λ_ex_ band appeared in the steady-state fluorescence spectra. For clearer data representation, the 735–745 nm range was removed from the spectra during plotting. To allow normalization of the spectra by area, the missing range was replaced with a linear function. Although this approach is rather crude for analysis, it was acceptable because area normalization was needed only to qualitatively demonstrate spectral shifts, and the areas themselves were not used for numerical analysis.

The image analysis and calculations were performed with Python v3.13.

### 2.7 Statistical Analysis

Pairwise comparisons between the data sets were performed using the unpaired two-tailed Welch’s *t*-test (assuming unequal variances) (scipy.stats module, Python). The *p*-values for significant differences (corresponding to Figures 1, 2, 6, and 8) are summarized in the Supporting Information (Figure S18 and Tables S6–S8).

**Figure 1:**
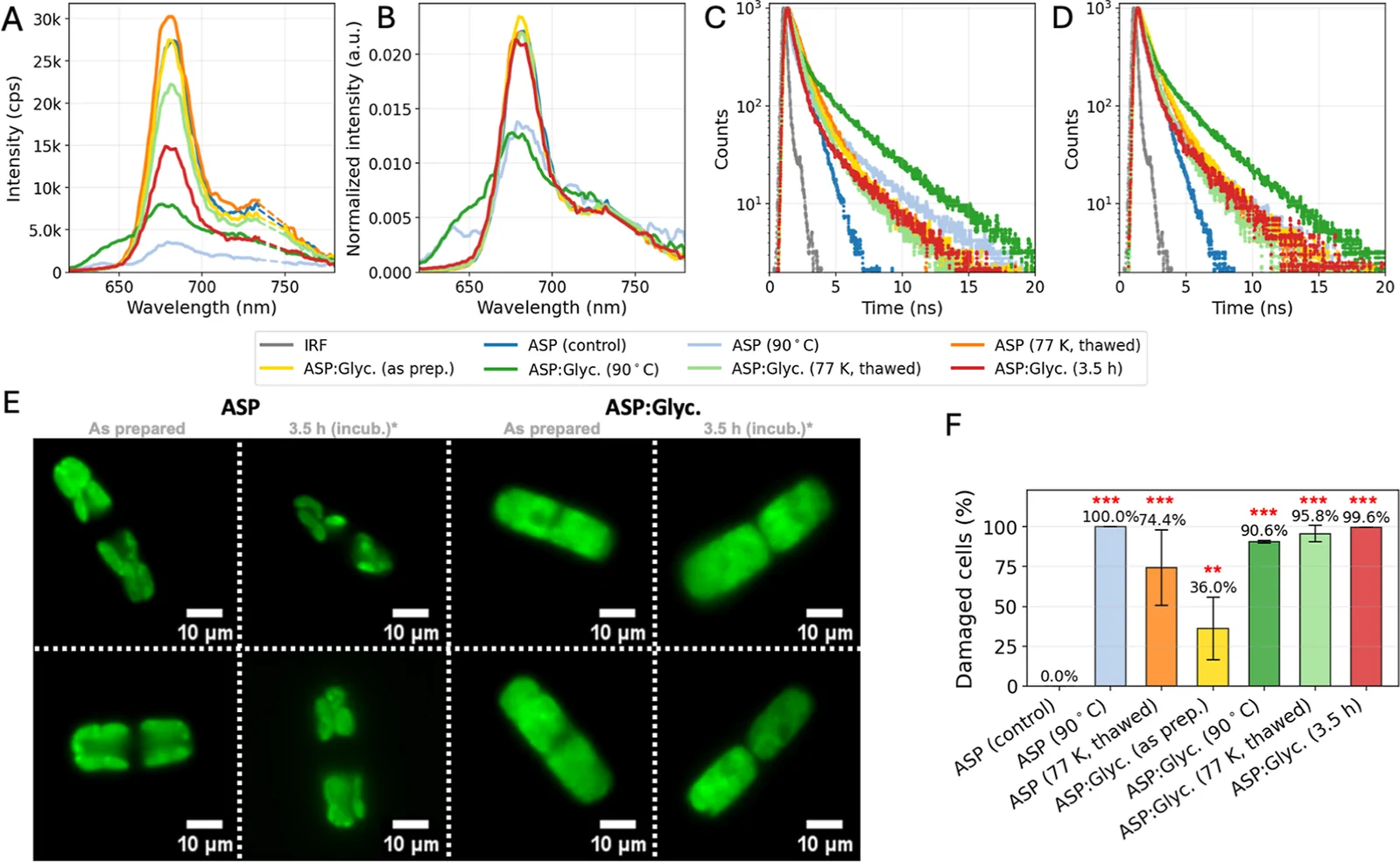
Effect of media and measurement conditions on the steady-state fluorescence emission spectra, time-resolved fluorescence decays, morphology of intracellular structures, and membrane integrity of *C. meneghiniana* cells. Panel A: emission spectra in absolute units; B: emission spectra normalized by area; C: fluorescence decays detected at 685 nm; D: fluorescence decays detected at 720 nm; E: fluorescence microscopy of cells mounted in ASP and ASP/glycerol mixture; F: percentage of damaged cells (with compromised membrane integrity). Wide-field fluorescence images were obtained using λ_ex_ = 405 nm. Membrane integrity was assessed using the fluorescence dye propidium iodide. The bars in F represent the area under the spectral curve calculated from 550 to 650 nm (Figure S4) and expressed as a percentage ± std, with cells in ASP media as the negative control and cells in ASP media held at 90 °C for 15 min as the positive control. The legend applies to A-D and F. The emission slits for emission spectra and fluorescence decay measurements were 5 nm. Dashed lines in A and B indicate regions with 2λ_ex_ (resulting from high scattering by the cell suspension and covering the 735–745 nm range) that were removed from the spectra to improve visualization and enable normalization of the spectra by area. Label “ASP/Glyc.” corresponds to the ASP/glycerol 2:3 ratio and is used to shorten the notations. For the same reason, “ASP/Glyc as prep.” denotes samples that were mixed immediately before measurements. *The 3.5 h incubation interval corresponds to the maximum time the cells remain mounted in ASP/glycerol medium before freezing for cryo-spectroscopy measurements (Figure S1). ***p < 0.001, **p < 0.01 indicate statistically significant differences between the control and treated groups in panel F. *p*-Values for pairwise comparison of treated groups are in Figure S18.

## 3 Results

### 3.1 Influence of Cryopreservation Media on Cell Viability and Its Manifestation in the Spectroscopic Features of *C. meneghiniana* Cells

A standard experimental workflow for cryogenic spectroscopy showing specific time intervals is given in Figure S1. The effect of cryopreservation media on the viability and functional state of cells in ASP and ASP/glycerol (2:3) media at key time checkpoints was assessed.

The spectral shapes and fluorescence intensities of cells in both ASP and ASP/glycerol media immediately after transfer to the cryopreservation medium are almost identical (Figure 1A). After 3.5 h in ASP/glycerol (the maximum time between sample preparation and freezing in a helium cryostat, Figure S1), an intensity decrease is observed (Figure 1A), with no changes in fluorescence spectral shapes (Figure 1B), which, according to ref,^38^ indicates cell viability and integrity. On the other hand, the fluorescence spectra of cells in ASP and ASP/glycerol media after a freeze–thaw cycle (Figure 1A,B), mimicking sample thawing in cryo-measurements during subsequent temperature increase to RT, also show no changes in spectral shapes compared to control cells in ASP at RT (Figure 1B), although thawing cells in ASP causes cell damage.^52^ Therefore, the spectral shape alone does not reflect cell viability or functional state.

Exchanging ASP for ASP/glycerol medium (2:3) leads to morphological changes in the chloroplasts of *C. meneghiniana*. In control *C. meneghiniana* cells in ASP, the chloroplasts are elongated, disc-shaped, located at the periphery of the cytoplasm, and clearly separated from each other by the central vacuole (Figure 1E). ASP/glycerol causes almost immediate swelling and degradation of chloroplasts and thylakoids (Figure 1E), indicating loss of normal function and disruption of the photosynthetic apparatus. As the chloroplasts disintegrate, the thylakoid content fills the entire cell volume, and no distinct structures are visible (Figure 1E, right panels). Chloroplast swelling is not caused by the decrease in salt concentration during ASP exchange for glycerol, as spectroscopic features are independent of salt concentration (Figure S5) and are instead caused by glycerol. An additional fluorometric viability assay with PI shows that the proportion of cells with lost membrane integrity (PI-positive) is about 36.0% immediately after transfer, and we cannot exclude the presence of glycerol-affected but PI-negative cells. Therefore, the PI-negative (∼64%) fraction is an upper bound on viability, but not a measure of functional PSII content or cell state. 3.5 h of cell exposure to ASP/glycerol results in more than 99% of damaged (PI-positive) cells (Figure 1F).

The fluorescence spectra of the positive control cells (ASP 90 °C and ASP/glycerol 90 °C, considered as “all dead”) show an additional band centered at 640 nm (Figure 1A,B), which is attributed to Chl *c* that has lost coupling to Chl *a* ^53–56^ and indicates destruction of the photosynthetic apparatus. The absence of the 640 nm band in the fluorescence spectra of cells mounted in ASP/glycerol (immediately after preparation and after 3.5 h) and subjected to the freeze/thaw cycle indicates no complete degradation of FCP complexes.

In contrast to the spectral shapes, the fluorescence decays of *C. meneghiniana* cells in ASP and ASP/glycerol are completely different (Figure 1C,D). Transferring the cells to ASP/glycerol leads to the appearance of a long component of approximately 2.5 ns (Table S1) and an increase in the average lifetime compared to cells in ASP. The characteristic lifetimes, pre-exponential factors (amplitudes), and average lifetimes of cells in ASP/glycerol are very similar to those of cells in ASP after a freeze–thaw cycle (Table S1). This indirectly indicates similar functional states of cells in ASP/glycerol and thawed cells and suggests a significant proportion of damaged cells.

The changes in fluorescence spectra and decays of cells mounted in ASP/glycerol are time-dependent (Figures 2A,B and S6). The absolute fluorescence intensity increases significantly immediately after the medium is exchanged to ASP/glycerol (2:3) and then subsequently decreases (Figure S6). Spectral changes are accompanied by an increase and then a decrease in the mean lifetime τ_AVG_, reaching a plateau after 1.5 h (Figure 2A,B). However, the spectral shapes almost perfectly coincide (Figure S6B), indicating that this does not reflect the structural or functional changes occurring in the thylakoid membranes.

**Figure 2:**
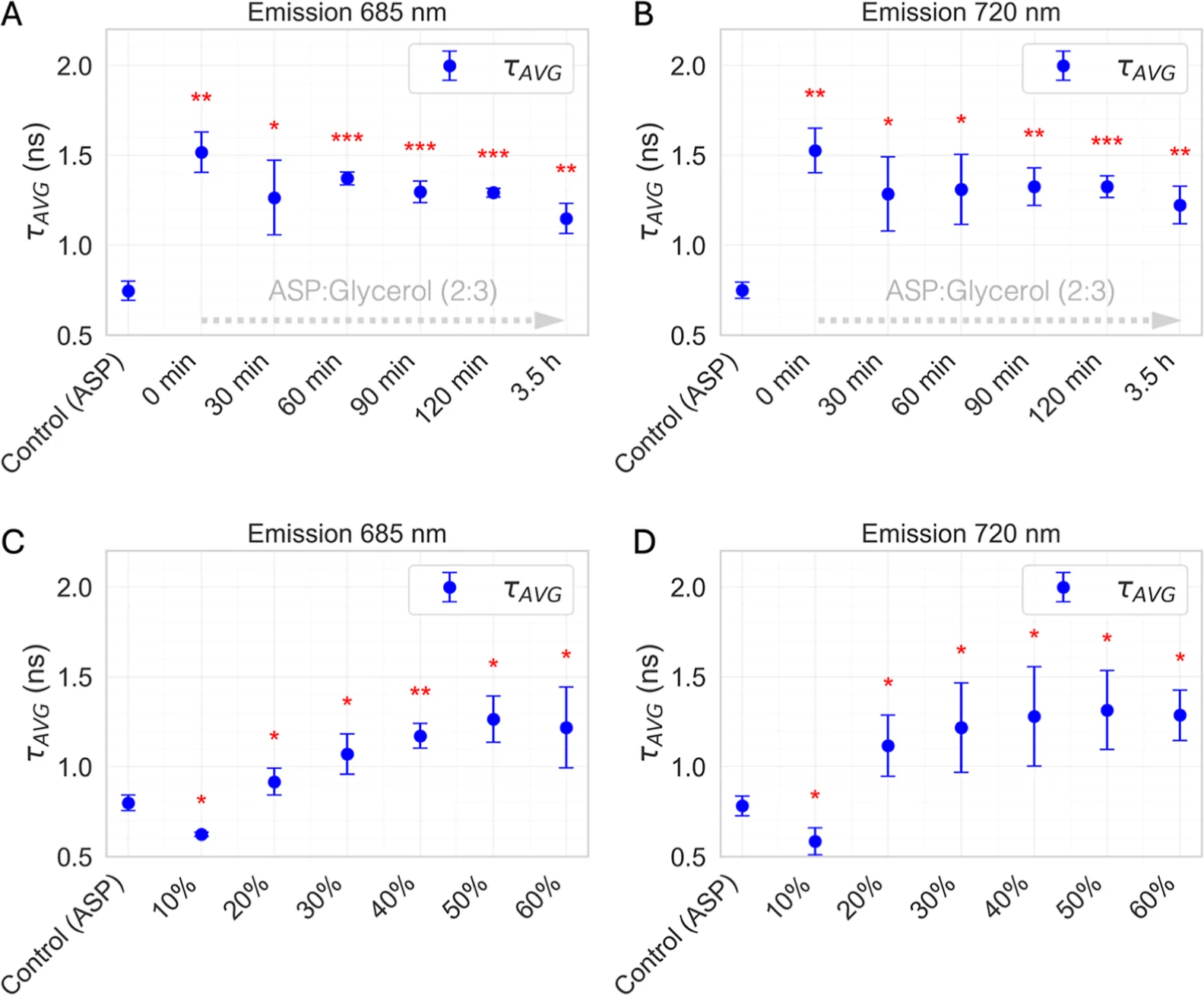
Concentration- and time-dependent effects of glycerol content in the measurement medium on the intensity-averaged lifetimes τ_AVG_ of *C. meneghiniana* cells. Panels A and B show the time-dependent changes of τ_AVG_ measured at 685 and 720 nm emission, respectively (in ASP/glycerol medium of 2:3 ratio). Panels C and D show the dependence of τ_AVG_ on the volume fraction of glycerol (3.5 h) in the ASP/glycerol medium measured at 685 and 720 nm emission, respectively. Measurements were performed at RT with λ_ex_ = 371 nm excitation. At least three independent measurements were performed. Data are presented as mean ± std. *p < 0.05, **p < 0.01, ***p < 0.001 indicate a significant difference between the control (ASP) and glycerol-treated groups. Numerical data on p-values are in Table S6.

Reducing the glycerol content does not eliminate the fluorescence changes induced in cells upon medium exchange (Figures 2C,D and S7). Replacing the ASP medium with media containing 10–60% (v/v) glycerol immediately alters fluorescence decay kinetics (Figure S7E,F), and this effect is not reversed after 3.5 h (Figure S7G,H). The fluorescence intensity is also affected (Figure S7A,C); however, the shapes of the spectra do not change significantly.

### 3.2 Electronic Excitation Dynamics Investigation in Intact-Frozen *C. meneghiniana* Cells: Cryogenic Steady-State and Time-Resolved Fluorescence Spectroscopy

Since glycerol affects the photosynthetic apparatus and causes cell death, further cryo-measurements were conducted in ASP medium, although increased scattering and reduced sample transparency can potentially affect the measurements.^57^

Figure 3 shows the steady-state fluorescence emission spectra and time-resolved fluorescence decays of *C. meneghiniana* cells measured in ASP medium at RT and cryogenic temperatures. After the initial RT measurements, the temperature was lowered to 16 K as quickly as possible, and spectra and decays were measured during stepwise heating from 16 K to RT. RT measurements after the freeze/thaw cycle were performed twice: first, immediately after the 16–270 K measurements, and second, approximately 12 h later (24 h after the experiment began) (see Figure S1). The latter served as a control for assessing loss of viability, with the assumption that all of the cells were dead.

**Figure 3:**
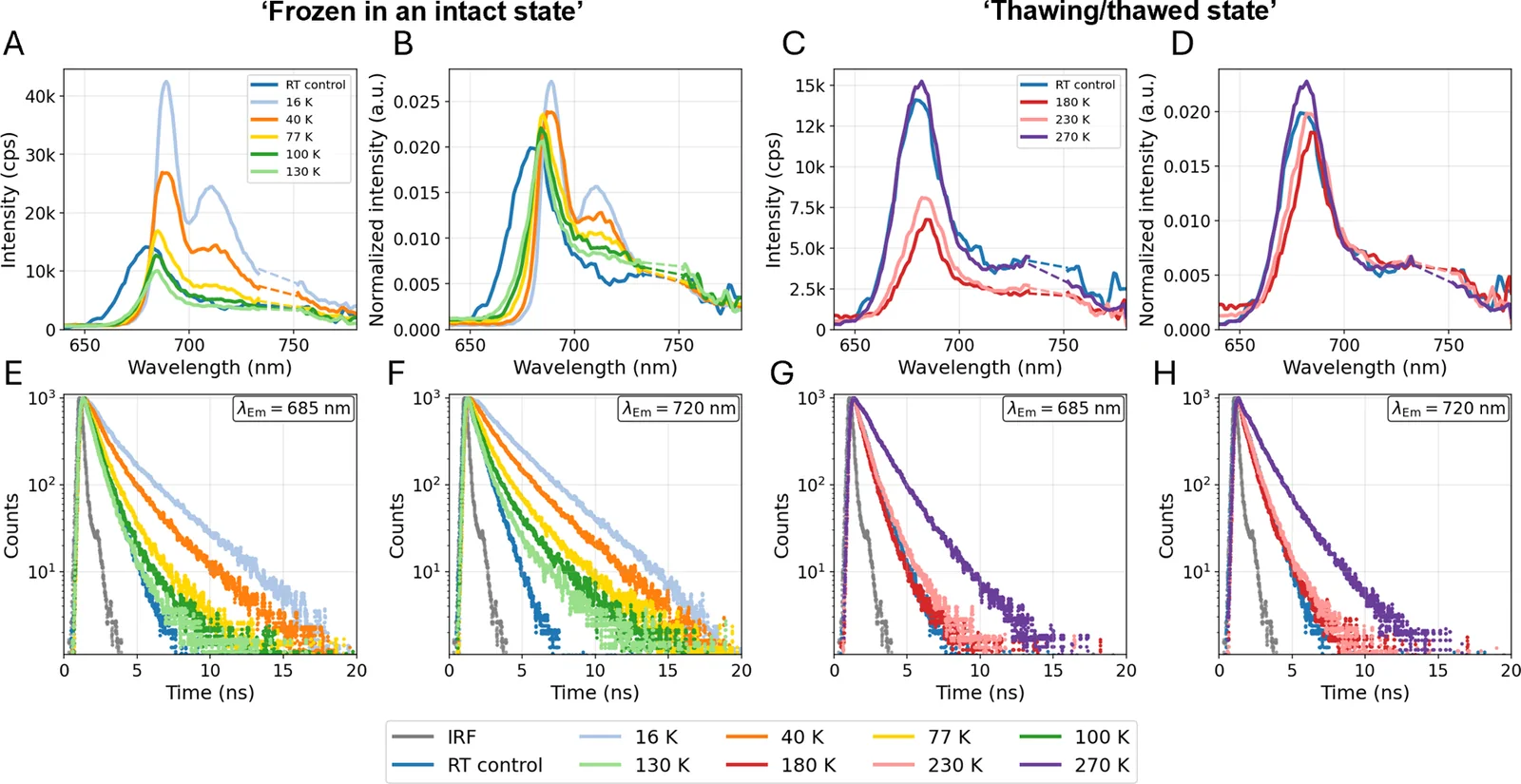
Temperature dependence of the steady-state fluorescence emission spectra and time-resolved fluorescence decays of *C. meneghiniana* cells measured in ASP medium at RT (prior temperature decrease) and at 16–270 K. Panels A and C show emission spectra in absolute units; B,D show emission spectra normalized by area; E and G show fluorescence decays detected at 685 nm; F and H show fluorescence decays detected at 720 nm. Panels A, B, E, and F correspond to the state of the cells considered “intact”, while C, D, G, and H correspond to the thawing/thawed state of the cells, considered the “nonviable” state. The dark blue curves represent measurements performed before the temperature decrease, referred to as “control”, and are provided in all panels for comparison. The emission slits for emission spectra and fluorescence decay measurements were 5 and 20 nm, respectively. Dashed lines in (A–D) indicate regions with 2λ_ex_ (resulting from high scattering by the cell suspension and covering the 735–745 nm range) that were removed from the spectra to improve visualization and enable normalization of the spectra by area.

The maximum of the fluorescence spectrum of intact cells measured before freezing (RT) is centered at 681 nm, while a second broad band of lower intensity spans 700–760 nm (Figure 3). The main band primarily represents PSII fluorescence, and the broad band corresponds to the vibrational sidebands. PSI is also expected to contribute to the broad band at 710–720 nm.^58^

At 16 K, there is a significant increase in fluorescence intensity and an 8 nm redshift of the 681 nm fluorescence band. A broad band centered at 711 nm becomes more pronounced compared to the RT control cells (Figure 3A,B). Increasing the temperature to 180 K causes a gradual blueshift of the band from 689 to 685 nm and a decrease in the intensity of the bands at 685 and 711 nm. At 230 and 270 K, the previously monotonic temperature dependence of the spectra is interrupted, with an increase in intensity and a further blueshift to 682 nm (Figure 3C,D). Further heating to RT did not affect the spectral shape. Similar results were observed 24 h after the experiment began.

The time-resolved fluorescence decays show a monotonic decrease in lifetime as the temperature increases from 16 to 130 K (Figure 3E,F). The fluorescence decays measured at 180 K are somewhat similar to those at RT (control) (Figure 3G,H), although the fluorescence peak is red-shifted at 180 K compared to RT (Figure 3C,D).

At 230 and 270 K, the monotonic changes in fluorescence kinetics are interrupted, and a slowing of the decays is observed (Figure 3G-H). This trend in fluorescence spectra and decays likely indicates degradation processes, which are completed at RT, with practically no changes observed over 12 h (within 24 h after the measurements began) (Figure S8).

Two-exponential functions were needed to fit the fluorescence decays at 685 nm for temperatures from 16 to 130 K and at RT (control, intact cell), while the decays measured at 180 K, 230 K, 270 K, and RT (after the freeze/thaw cycle) required a three-exponential model (Figure 4A). As shown in Figure 4B, a three-exponential model was also required to fit the decays measured at 720 nm at 100 and 130 K.

**Figure 4:**
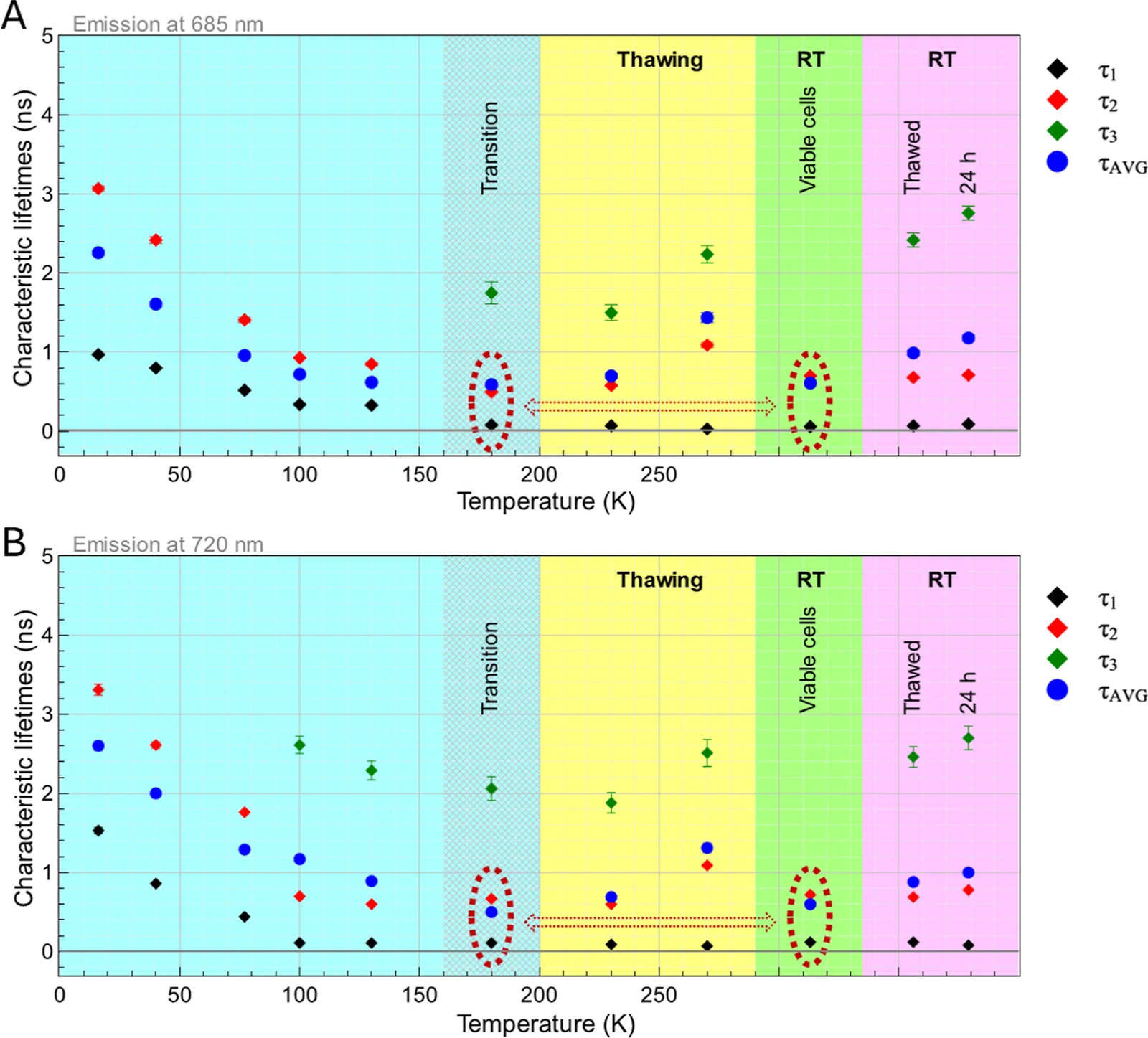
Temperature dependence of the characteristic lifetimes. Panel A:Characteristic lifetimes calculated for fluorescence decay kinetics measured at 685 nm; B: those measured at 720 nm. The cryogenic temperatures ranged from 16 to 270 K. Green and pink areas indicate room temperature measurements performed on the same sample before and after cryogenic measurements, respectively. “Thawed” refers to the measurement performed on the same day as the measurements at other temperatures, while “24 h” indicates the measurement performed 24 h after the measurements started. The red arrow indicates the similarity of the characteristic lifetimes at 180 K and at RT for intact cells. Five independent measurements were performed in ASP medium (without glycerol), and the data shown correspond to one representative measurement. The decays were fitted with either two or three exponential functions, depending on the fitting quality. The emission slit for the fluorescence decay measurements was 20 nm.

The fast and slow components of fluorescence decay measured at 16 K are 0.97 and 3.07 ns for emission at 685 nm and 1.53 and 3.31 ns for emission at 720 nm. These time scales are significantly longer than those measured at RT with viable cells, as expected.

Although the characteristic lifetimes (both τ_*i*_ and τ_AVG_) decrease monotonically in the 16–180 K temperature range and increase during the thawing phase, the pre-exponential factors (amplitudes) vary less, accounting for approximately 60–70% and 30–40% for the fast and slow components, respectively (Table S2). The contribution of the third component remains within 2–4%. The fractional contributions of the fast and slow components for emission at 685 nm are similar at low temperatures up to 130 K, but above 180 K, the slow component becomes dominant (Table S2). For emission at 720 nm, the fractional contribution of the slow component is about 60% across the entire temperature range.

There are marked differences between the data for 16–130 K and those corresponding to further heating of the cells. First, the decays at 685 and 720 nm are clearly slower than RT control results. Second, there is no pronounced third component. Third, the temperature dependence of the lifetimes is monotonic. In contrast, the 180 K data already differ, as the initial part of the decay at 685 nm is even faster than that in the RT control measurements, and a third component is needed to fit the kinetics, which is not the case for the 130 K and RT control data. Further increases in temperature break the monotonic temperature dependence of the average lifetime.

Considering all this, we hold that measurements performed in the temperature range from 16 to 130 K can be considered measurements of intact-frozen cells; 180 K data represent a transition state, while the temperature range from 230 to 270 K corresponds to sample thawing and should not be used to describe the photosynthetic machinery of intact cells of *C. meneghiniana*. However, since measurements with a lower temperature step were not performed, the 130 K temperature is not a strict, but rather a conservative upper bound of the “intact” state.

### 3.3 Cryogenic Spectroscopic Measurements of *C. meneghiniana* Cells in ASP/glycerol (2:3)

Additional measurements of *C. meneghiniana* cells in ASP/glycerol (2:3) medium were performed, and a comparison of the spectroscopic characteristics of cells in ASP and ASP/glycerol is shown in Figure 5. Since 99% of cells in ASP/glycerol (2:3) were found to be dead, only a general evaluation of differences is provided, without detailing the origin of spectral changes, as the photosynthetic machinery of dead cells is not within the scope of this study.

**Figure 5:**
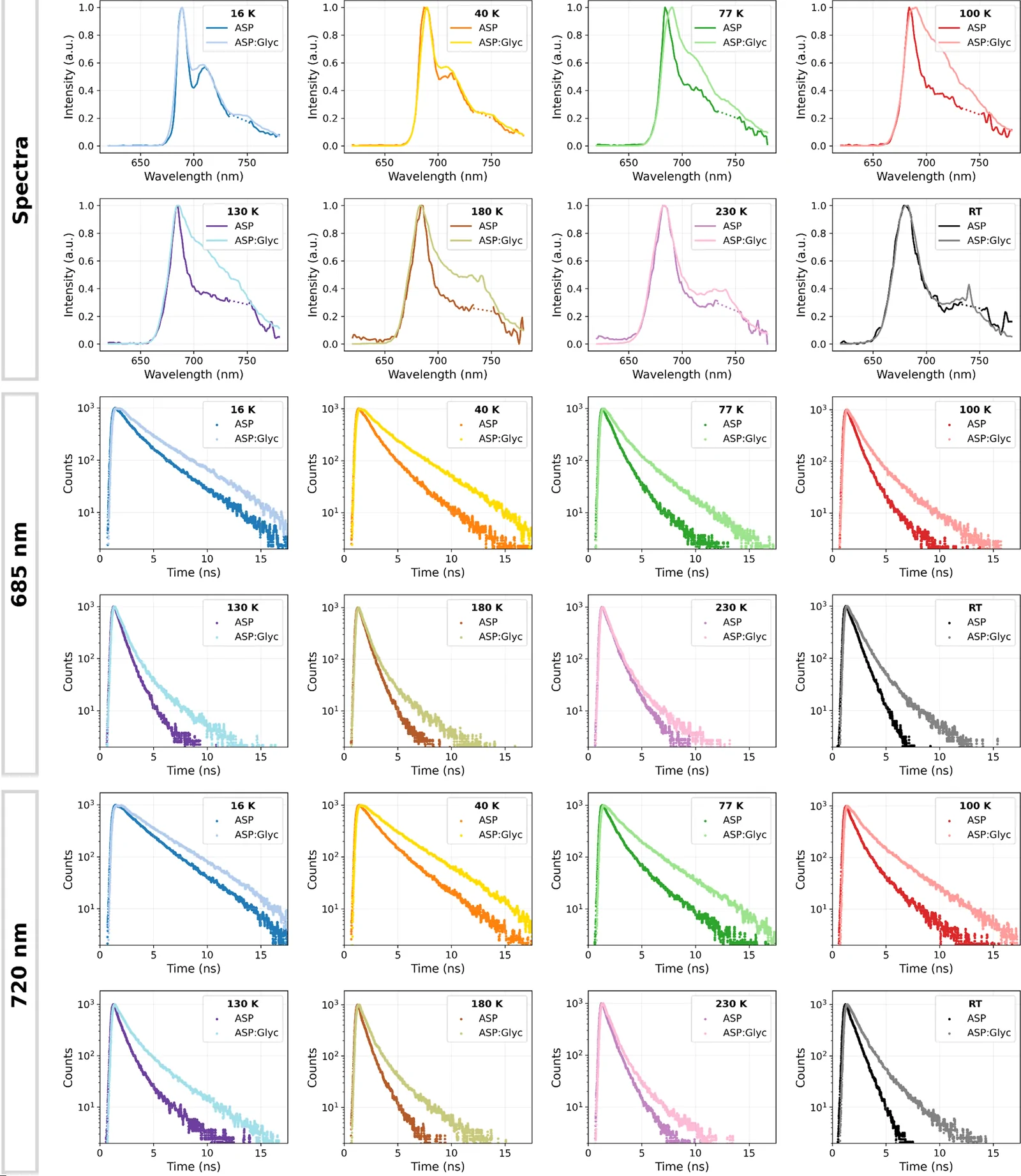
Comparison of fluorescence spectra, normalized to the maximum intensity, and decays of *C. meneghiniana* cells measured at cryogenic temperatures in ASP and ASP/glycerol (2:3) media. Excitation wavelength was λ_ex_ = 371 nm. Dashed lines in spectra of cells exposed to ASP indicate regions with 2λ_ex_ (resulting from high scattering by the cell suspension and covering the 735–745 nm range) that were removed from the spectra to improve visualization. The emission slits for emission spectra and fluorescence decay measurements were 5 and 20 nm, respectively.

The relative contribution of the 700–750 nm broad band to the fluorescence spectra of cells mounted in ASP/glycerol medium is significantly higher compared to cells measured in ASP medium, with a more pronounced effect observed at temperatures from 40 to 180 K. In addition, glycerol induces a redshift of the 685 nm band at temperatures from 40 to 100 K. The fluorescence decays at all temperatures and at both wavelengths are characterized by significantly increased average fluorescence lifetimes, indicating dramatic changes in the photosynthetic apparatus caused by a 60% exchange of ASP with glycerol. The increased intensity of the red band at low temperatures is reminiscent of the fluorescence of FCP aggregates, which have been shown to exhibit a red component, which blueshifts at lower temperatures.^16^ This suggests that at least some FCPs likely decouple from PSII under glycerol treatment and form variously sized aggregates.

### 3.4 Cryogenic Measurements of *C. meneghiniana* Cells under Treatment with DCMU and FCCP

To analyze the contribution of the constituents of the photosynthetic machinery to whole-cell fluorescence, the workflow described above was repeated with *C. meneghiniana* cells treated with either DCMU, an inhibitor of electron transfer from PSII to the plastoquinone pool, or FCCP, a protonophore that affects not only the *trans*-thylakoidal proton gradient but also uncouples mitochondrial oxidative phosphorylation.

#### 3.4.1 *C. meneghiniana* Cells Treated with 20 μM DCMU

The temperature dependence of the steady-state fluorescence spectra and fluorescence decays of *C. meneghiniana* cells upon 20 μM DCMU treatment is shown in Figure S9. Similarly to untreated cells, there is a gradual blueshift in spectra from 691 to 686 nm when heating from 16 to 130 K. However, in contrast to the untreated cells, for DCMU-treated cells, a broadening of the band at 680–690 nm occurs, more likely due to the increased contribution of the 695 nm component and decreased impact of 680 nm at temperatures ranging from 40 to 130 K (Figure S9A,B). The band centered at 712 nm is pronounced at 16 and 40 K and changes similarly to the case of the untreated cells. At temperatures of 180 K, 230 K, and 270 K, a subsequent blueshift from 684 to 682 nm occurs, accompanied by an increase in fluorescence intensity by almost two times (Figure S9C,D), a trend that is also characteristic of untreated cells.

In terms of the time-resolved fluorescence measurements, the general trends in the fluorescence decays at cryogenic temperatures are more or less similar to those of untreated cells and demonstrate monotonic changes in the temperature range from 16 to 130 K, with the temperature of 180 K being a “transition” point (Figure S9E,F). There is almost complete coincidence of the fluorescence decays at 180 and 230 K and an increase in the average lifetime at 270 K. The fluorescence decays of thawed cells also coincide (Figure S10). A significant difference in the fluorescence decays of DCMU-treated cells as compared to untreated cells is observed at RT prior to cryo-measurements (Figure S9E–H) and consists of a significant increase in the fluorescence decay time.

The comparison of the numerical data obtained from fitting the fluorescence decays of DCMU-treated *C. meneghiniana* cells is presented in Figure 6 for the intensity-averaged lifetimes. Numerical values can also be found in (i) Figure S11a,b and Table S2 for untreated cells; and in (ii) Figure S11c,d and Table S3, for DCMU-treated cells.

**Figure 6:**
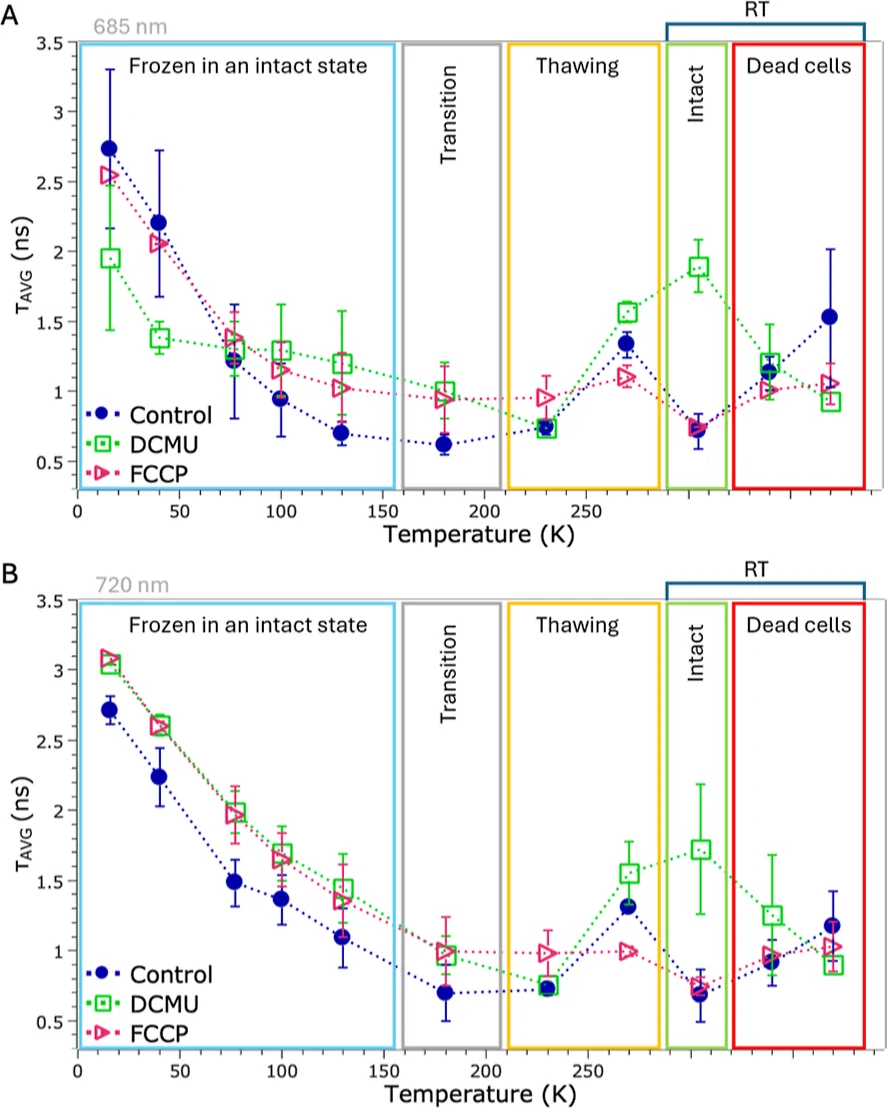
Temperature dependence of the averaged fluorescence lifetimes of untreated cells and cells treated with either 20 μM DCMU or 250 nM FCCP and measured at 685 nm (A) and 720 nm (B). The cryogenic temperatures ranged from 16 to 270 K. Green and red rectangles indicate room temperature measurements performed on the same sample before and after cryogenic measurements, respectively. The “Thawed” label refers to the measurement performed on the same day as the measurements at other temperatures, while the “24 h” label indicates the measurement performed 24 h after the measurements with the cryostat started. Five independent measurements were performed, and the data shown correspond to the mean ± std. The decays were fitted with either two or three exponential functions, depending on the fitting quality. The emission slit for the fluorescence decay measurements was 20 nm. The *p*-values of pairwise comparison between Control/DCMU, Control/FCCP, and DCMU/FCCP are given in Table S7.

In contrast to untreated cells, three exponential functions were required to fit the experimental data upon DCMU treatment in almost all cases. In addition, for low-temperature measurements, fast components (τ_*1*_) were missing (or not resolved due to experimental setup limitations), while slow components of 2–3 ns were present with a high fractional contribution (e.g., τ_*3*_^16 K^ = 3.2 ns, *f*_*3*_^16 K^ = 67%, 685 nm emission, Table S3), which, however, resulted in intensity average lifetimes comparable to those of untreated cells (Figure 6A). The contribution of τ_*3*_ gradually decreased to 15% with the temperature increase to 230 K, but subsequent heating to 270 K led to an increase in its fractional contribution. The values of τ_*1*_ and τ_*2*_ as well as their fractional contributions to the fluorescence decays measured at 685 nm emission remained practically unchanged in the temperature range 40 K–180 K. This resulted in an almost constant value of the average lifetime (685 nm emission) in this temperature range (Figure 6A) for DCMU-treated cells. Starting from 77 K and up to 180 K, the average lifetimes of the fluorescence decays measured at 685 nm of DCMU-treated cells surpass the values of the average lifetimes for untreated cells.

In contrast to 685 nm emission, the average lifetimes of the decays measured at 720 nm of untreated and DCMU-treated cells have the same trend in temperature dependence for the whole temperature range (except for RT measurements prior to cryo-measurements), although the magnitude of the τ_AVG_ is higher for DCMU-treated cells (Figure 6B). The latter is more likely due to the relatively high fractional contribution of the slow component (τ_3_) observed for the whole temperature range (Table S3).

Regarding the RT measurements of the thawed DCMU-treated cells, as mentioned above, the spectral shapes and the fluorescence decays practically coincide (Figure S10); however, a slight decrease in the average lifetime is observed 24 h after the measurements began (Figure 6), more likely related to the mixing of the cells (similar to untreated cells).

#### 3.4.2 *C. meneghiniana* Cells Treated with 250 nM FCCP

The steady-state and time-resolved fluorescence measurements of the suspension of *C. meneghiniana* cells treated with 250 nM FCCP are shown in Figures S12 and S13. As shown in Figure S12A–D, the band centered at 691 nm shifts to 685 nm as the temperature increases from 16 to 130 K. As in DCMU-treated cells, this band broadens due to an increased contribution from the 695 nm component and a decreased contribution from the 685 nm component. As in untreated and DCMU-treated cells, a further blueshift to 682 nm occurs when the temperature is raised to 270 K. However, unlike untreated and DCMU-treated cells, no increase in fluorescence intensity is observed at 270 K (Figure S12C).

Time-resolved fluorescence measurements of FCCP-treated cells show monotonic behavior in the fluorescence decays measured at 685 and 720 nm emission in the temperature range from 16 to 130 K, resembling those of untreated and DCMU-treated cells. The fluorescence decays at 180, 230, and 270 K almost coincide when measured at both 685 and 720 nm (Figure S12G,H), which is completely different from the decays observed in untreated and DCMU-treated cells.

The results of the fluorescence decay analysis for FCCP-treated cells are presented in Table S4, with comparisons to untreated and DCMU-treated cells shown in Figures 6 and S11. As with DCMU-treated cells, fitting the fluorescence decays required three exponential components, except for measurements at 16 and 40 K at 685 and 720 nm, and at RT (viable cells) at 685 nm. These exceptions were well described by two exponential functions, lacking the fastest component. However, unlike DCMU-treated cells, the temperature dependence trends of the average lifetimes (for both 685 and 720 nm measurements) in FCCP-treated cells resembled those of untreated cells. Nevertheless, the average lifetimes of FCCP-treated cells are statistically higher than those of untreated cells at 16–230 K for 720 nm emission and 77–230 K for 685 nm emission (Figure 6). Notably, the average lifetimes of the fluorescence decays in FCCP-treated cells at 270 K are significantly lower than those measured for untreated and DCMU-treated cells, indicating that dissipation of the proton gradient cancels the significant increase in average lifetimes observed in untreated and DCMU-treated cells at 270 K.

#### 3.4.3 FCCP Effects on the Cells Undergoing Freezing/Thawing Cycle

To interpret the unexpected increase in fluorescence intensity and average lifetimes observed at 270 K upon thawing for untreated and DCMU-treated cells, as well as the cancellation of this effect by FCCP treatment, time-dependent changes in fluorescence spectra and fluorescence decays of *C. meneghiniana* cells upon thawing were investigated. For this purpose, several separate vials with cell suspensions of untreated cells and cells treated with 250 nM FCCP were frozen with liquid nitrogen and then removed and measured one by one upon thawing. The complete data set related to untreated cells is shown in Figure S14, and that related to 250 nM FCCP-treated cells is shown in Figure S15. The initial and final phases of thawing are shown in Figure 7 for untreated and FCCP-treated cells, respectively.

**Figure 7:**
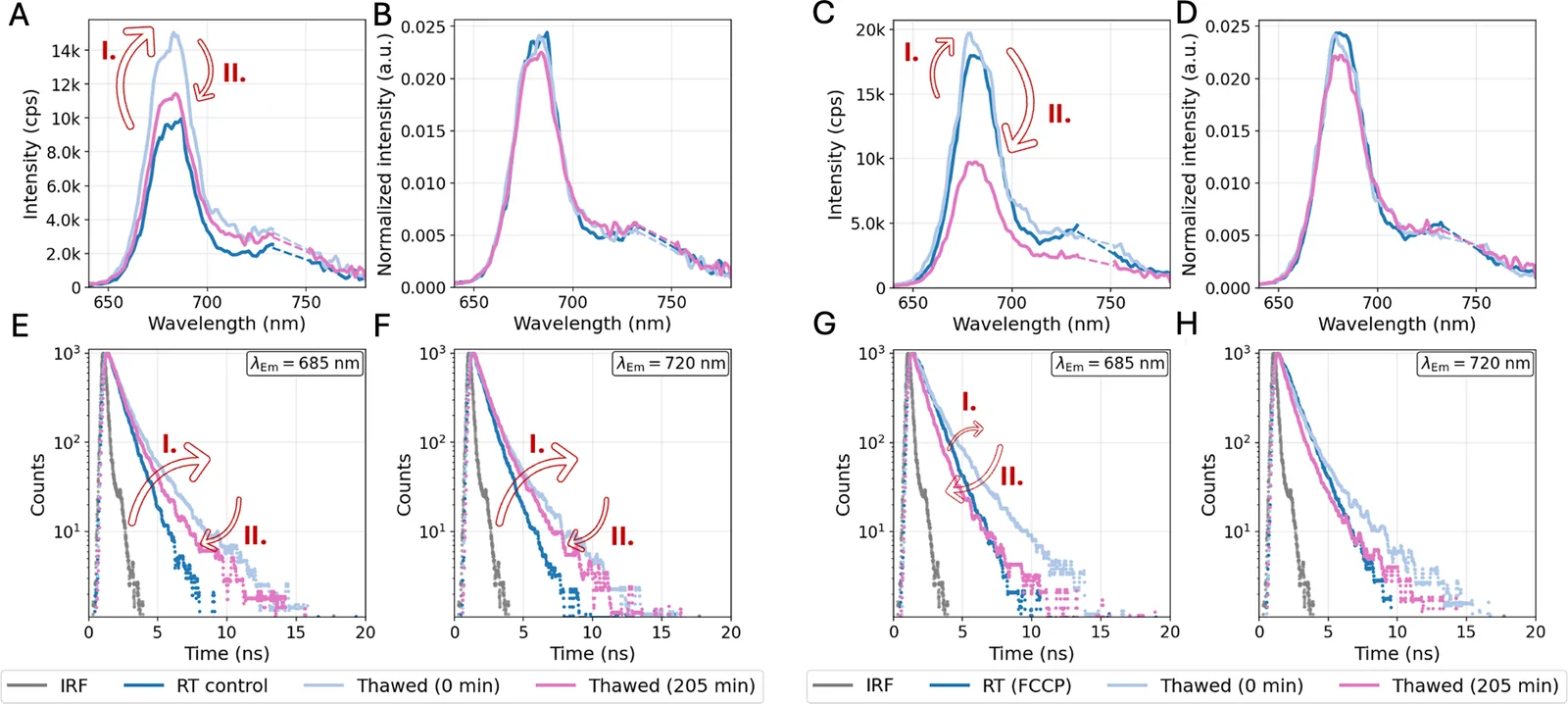
Steady-state fluorescence emission spectra (A,B) and time-resolved fluorescence decays (C,D) of untreated cells *C. meneghiniana* (A, B, E, F) and treated with 250 nM FCCP (C, D, G, H) measured before the freezing (at 77 K)-thawing cycle and over the time after thawing: (A, C) emission spectra in absolute units, (B, D) emission spectra normalized by area, (E, G) fluorescence decays detected at 685 nm, and (F, H) fluorescence decays detected at 720 nm. The measurements were performed at RT at λ_ex_ = 371 nm. Numbers I and II indicate the initial and the final phases of thawing, respectively. The arrows indicate the direction of changes, while their lengths show the relative changes in the transitions from the initial state to phase I compared to the transition from phase II to phase I.

As follows from Figures 7, S14, and S15, in both cases, an increase in the fluorescence intensity accompanied by an increase in the average lifetime occurs during the initial phase of thawing (denoted as phase I in Figures 7, S14, and S15) and is followed by a subsequent decrease in the final phase of thawing (phase II). However, in untreated cells, the intensities and the lifetimes (phase II) are higher than before thawing (Figure 7), while in FCCP-treated cells, the fluorescence intensity and fluorescence decay average lifetimes drop and are lower in magnitude than those of the cells before freezing. This effect is demonstrated more clearly through average lifetimes calculated for the fluorescence decays measured over time upon thawing (Figure 8). As follows from Figure 8, fluorescence intensity and fluorescence average lifetime increase at 270 K, as observed in Figure 3, occur due to cell damage and are similar to the average lifetime increase shown in Figures 7 and 8, while FCCP treatment cancels this effect.

**Figure 8:**
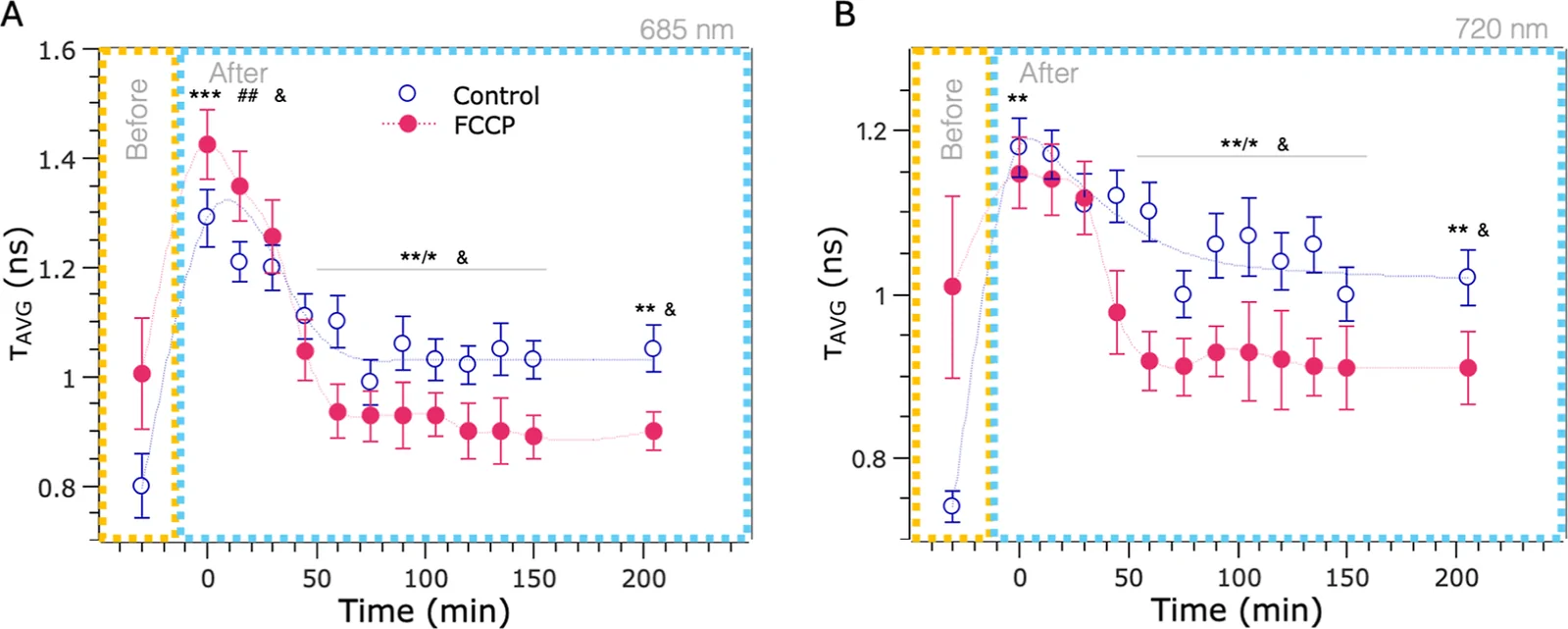
Temporal changes of the average lifetime of the fluorescence decay kinetics measured at 685 nm (A) and 720 nm (B) of *C. meneghiniana* cells undergoing freezing/thawing cycle. The measurements were performed on unaffected cells and cells incubated with 250 nM FCCP for 10 min in the dark prior to freezing. The vials with cell suspensions were frozen by immersion in liquid nitrogen and kept at 77 K for 15 min. The dotted lines are shown to demonstrate the trends in τ_AVG_ changes over time. Data are presented as an average of three independent experiments ± std. ***p < 0.001, **p < 0.01 indicate statistically significant difference between untreated (Control) cells after thawing (AT) versus untreated (Control) cells before freezing (BF), ^##^ *p* < 0.01, ^#^ *p* < 0.05, FCCP-treated (AT) versus FCCP-treated (BF), and ^&^ *p* < 0.05 for FCCP-treated (AT) vs untreated (Control) (AT). Corresponding *p*-values for all pairwise comparisons between groups are given in Table S8.

## 4 Discussion

### 4.1 Spectroscopic Signatures of Structural Disorganization of Cellular Membranes in the Presence of Glycerol: False Cryoprotection

Glycerol is one of the most common components for spectroscopic cryomeasurements since it helps to avoid water crystal formation and thereby unwanted scattering. The media for cryo-measurements usually contain 30 to 60% glycerol (v/v), which, however, cannot be considered as a physiological environment. Although this fact can be mostly neglected for the measurements of isolated protein complexes, whole-cell measurements should be conducted with greater caution. However, none of the studies performed on diatoms provide any viability assessment,^21,22,38,39^ with rare attempts to confirm the cell intactness based on the coincidence or similarities in the shape of the fluorescence spectra.^38^ Such data are often left without significant attention, mentioned in passing in the methods, or are not even shown.

Here, we demonstrate that ASP media exchange to the ASP/glycerol mixture induces continuous changes in the fluorescence decays of the cells, while spectral shape and maximum position remain almost unchanged (Figure 1, A–D). Fluorescence microscopy data indicate very fast structural changes of thylakoid membranes (Figure 1E). Viability assay with PI reveals more than a third of cells being damaged (Figure 1F) even immediately after glycerol addition. Since an increase in PI fluorescence occurs only when it penetrates the cell, we assume that the outer cell membrane of *C. meneghiniana* exposed to ASP/glycerol is compromised, likely due to the swelling of the organelles, observed via fluorescence microscopy. Considering the possible changes in the photosynthetic apparatus, as Chl *c* is not released (no 640 nm band in fluorescence spectra of cells in ASP/glycerol mixture, Figure S4), FCP complexes should remain intact. In addition, complete disassembly of PS cores can be excluded, as no spectroscopic features of free Chl *a*—such as an additional shoulder around 665–675 nm—are observed. This indicates that the changes in the fluorescence decays upon media exchange to ASP/glycerol occur either due to alterations in the environment of the photosynthetic apparatus or as a result of changes in the interconnectivity of complexes.

A destructive effect of glycerol at concentrations ranging from 0 to 50% (v/v %) on the structural connections of phycobilisomes to the photosystems was previously observed in steady-state fluorescence spectroscopy studies of *Spirulina platensis* cells at 77 K.^40^ It was shown that phycobilisome fluorescence increases while PSII fluorescence decreases in cells as the glycerol concentration increases, whereas no changes occur in isolated thylakoid membranes. These cellular-level changes were thought to result from structural alterations in phycobilisomes and/or detachment of phycobilisomes from the membrane, caused by glycerol penetrating the thylakoid membrane and weakening hydrophobic interactions.^40^ Glycerol was also shown to inhibit micellization of sodium dodecyl sulfate by reducing solvophobic interactions,^59^ an effect that may significantly alter the membrane stability. On artificial dipalmitoylphosphatidylcholine bilayers, glycerol was shown to increase the magnitude of the repulsive forces between the bilayers.^60^

A similar effect of glycerol on lipid membranes may explain the changes observed in this study. Weakening of hydrophobic interactions between the lipids of the thylakoid membranes may affect the existing connections between thylakoids^61,62^ and the diffusion of electron transfer components between the photosystems. Additionally, alterations in the membrane fluidity caused by glycerol may affect the diadinoxanthin-diatoxanthin cycle,^63^ which in turn influences nonphotochemical quenching and thus fluorescence. It was also demonstrated that lipids may play a key role in the stabilization of FCP oligomers in *P. tricornutum* and *Ch. gracilis*,^64–66^ indicating that glycerol-induced lipid bilayer alterations may affect the structure of the light-harvesting apparatus.

In diatoms, PSI is located mainly in the stroma-exposed thylakoids while PSII is more abundant in the core of the stack,^61^ and, therefore, under detergent treatment, PSI (together with cytochrome (Cyt) b_6_f complexes, also located in the peripheral regions) extraction occurs at lower detergent concentrations than PSII. The latter allows expecting a higher susceptibility of PSI/Cyt b_6_f to glycerol action at the initial state of media exchange. However, loss of the connection between FCP closely associated with PSI should lead to an increase in fluorescence at 720 nm.^58^ This is not observed (Figure 1B); therefore, PSI-FCP most likely remains intact, and, since its fluorescence decays rapidly at RT, it is difficult to draw any conclusions about PSI. Therefore, higher τ_AVG_ values during the initial phase of glycerol action, followed by a moderate decrease, are associated with initial membrane reorganizations and are primarily related to PSII and FCP (Figure 2).

After the exchange of ASP to ASP/glycerol, a very long decay component appears (τ_3_, Table S1). The slowing of the fluorescence decays and the increase in fluorescence may indicate that more PSII RCs become closed due to impaired electron transfer resulting from osmotic effects such as swelling. This would lead to an increase in the fluorescence yield and a corresponding increase in the average lifetime,^67^ which disappears after some time due to temporary restoration of the electron transfer activity and thus PSII-RC reopening. However, a similar effect on fluorescence can be caused by the disconnection of FCPs as separate units or small clusters, as they have long fluorescence lifetimes at RT.^16^ Although the appearance of τ_3_ is a signature of morphological changes in chloroplasts and loss of cell viability, it could not be used for quantitative assessment of the portion of damaged cells. As shown for samples with 99% dead cells (Table S1), the fluorescence decays are characterized by three exponentials, not just one τ_3_. Therefore, quantitatively disentangling the contributions from dead and intact cell populations to the fluorescence decay, when at least 36% of the cells are damaged, is very complicated and challenging.

Further evolution of τ_AVG_ more likely indicates that FCPs become disconnected from PSII and tend to aggregate within the membrane. Since the aggregates may contain some complexes that are in a quenched state,^16^ this could be the reason for the presence of fast components in the fluorescence decays (Table S1 for ASP/glycerol 3.5 h and Figures 2 and S6). Similarities in the temperature dependence trends and τ_AVG_ values of fluorescence decays at 720 nm for ASP/glycerol-affected cells (3.35 ns at 15 K; 2.47 ns at 77 K; 1.10 ns at 180 K, Table S5) and for the red component of FCPb aggregates (2.48 ns at 15 K; 2.18 ns at 80 K; 0.81 ns at 180 K, calculated based on^16^) are in line with FCP aggregation in affected cells. However, the aggregates are expected to be heterogeneous in size, which explains the nonexponential decays. Such aggregation can be driven by the electrostatic interaction of proteins and glycerol molecules and preferential orientation of hydrophobic domains of the proteins toward each other rather than to glycerol.^68^ As mentioned in Section 2.3, an increase in the fluorescence intensity of the red band at low temperatures (Figure 5) also supports the hypothesis that decoupling of some FCPs from the PSII core and formation of FCP aggregates likely occurs due to the presence of glycerol, as shown in Figure 9.

**Figure 9:**
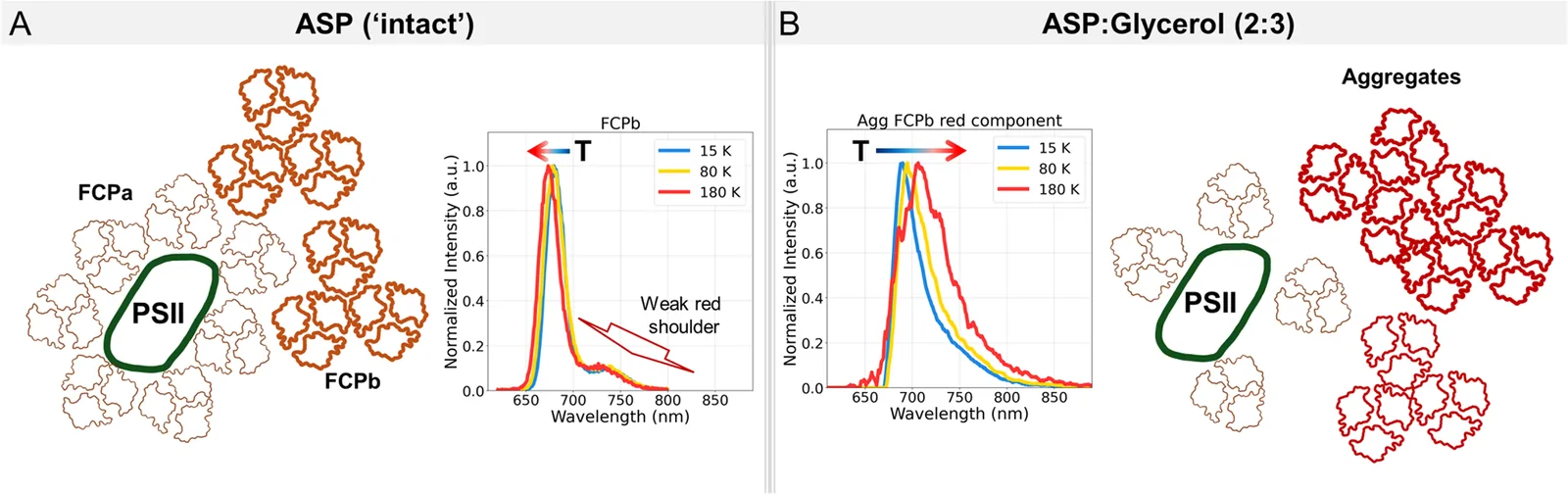
Proposed changes in the PSII supercomplex induced by exposure to glycerol. Panel A shows a schematic model of the intact PSII supercomplex of *C. meneghiniana* and the temperature-dependent spectra of isolated FCPbs. Panel B shows the scheme of the disruption of the PSII supercomplex, the formation of far-red emitting aggregates of FCPs (colored burgundy), the disassembly of peripheral FCPs from the PSII supercomplex, composed of the core (marked in green) and minor antenna, and the temperature dependence of the corresponding red components of the fluorescence spectra of FCPb aggregates. Spectral data for FCPbs and the red components of FCPb aggregates (obtained by multivariate curve resolution analysis of streak camera data) were taken from ref 16. Arrows in the spectra indicate the shift of the fluorescence maxima position with increasing temperature. The model of peripheral FCP arrangement around the PSII core is arbitrary, as currently the structural data show only the location of minor antenna complexes for *C. meneghiniana*.^10^

To estimate the generalizability of the results, we screened the effects of glycerol at RT of four different photosynthetic organisms: *Chlorella vulgaris* (green alga), *Isochrysis galbana* (Haptophyte), *Nannochloropsis oceanica* (Eustigmatophyte), and *P. tricornutum* (pennate diatom) (Figure S16). According to the RT data, as with *C. meneghiniana*, the spectral shape does not change (except for *P. tricornutum*), although the intensity changes (increases at the initial phase of medium exchange and subsequently decreases in *I. galbana* and *P. tricornutum*; decreases in *C. vulgaris* and *N. oceanica*). The trend in τ_AVG_ changes in *I. galbana* and *P. tricornutum* and also resembles that of *C. meneghiniana*: the kinetics become slower, indicating that the photosynthetic apparatus of all species is significantly affected by glycerol. *C. vulgaris* and *N. oceanica*, in contrast, are characterized by shorter τ_AVG_, indicating that glycerol induced quenching. The results indicate that glycerol affects the spectroscopic properties of all the analyzed species; however, the mechanisms of its effects are complex and species-specific and require additional targeted investigation.

Therefore, we conclude that data on cell measurements in the ASP/glycerol medium reported in the literature should be interpreted with caution, as they likely represent a mixed state that includes intact cells, dead cells, and cells in a transitional state. Unfortunately, it is often difficult to estimate the proportion of intact cells in reported data. Therefore, additional research, such as using a streak camera for improved time resolution, may help clarify alterations in the photosynthetic apparatus under glycerol treatment, which could help with reconsideration of already published data.

### 4.2 Specificity of Temperature Dependence of Electronic Excitation Dynamics in *C. meneghiniana* Cells Frozen in the “Intact” State

Most studies are conducted on isolated PSs, FCPs, PS-FCPs, or cells exposed to glycerol, which disrupts electron transfer through components of the photosynthetic apparatus and, therefore, cannot reflect the true state of the complex processes occurring at the whole-cell level. Low-temperature measurements performed in ASP media provide valuable information on the photosynthetic machinery of cells frozen in an “intact” state, revealing the changing contributions of different parts of the supercomplexes and components of electron-transfer chains at temperatures ranging from 16 to 130 K (Figures 3 and 4).

However, there are several issues that should be taken into account when spectroscopic measurements at cryogenic temperatures are conducted on cells mounted in ASP media.

ASP media contains 86 mM NaCl, which defines the freezing behavior of the solution. During freezing, solutes, together with cells, are expelled from the crystals that form during the freezing and are concentrated in the liquid phase—freeze-concentrated solution (FCS), which collects at the ice grain boundaries.^69^ FCS remains liquid until the eutectic temperature, at which the system freezes completely.^70^ The eutectic temperature for the system water/NaCl is 251.8 K. However, it can vary since ASP contains other salts and cells. Additionally, evidence exists that FCS may stay liquid even at temperatures below the eutectic temperature,^71^ specifically in diluted solutions, which ASP media can be attributed to.

FCS is characterized by higher salinity, and this can affect the cells.^72^ However, *C. meneghiniana* belongs to euryhaline diatoms with high salinity tolerance, enabling its successful survival in high salinities.^73^ Variation of NaCl concentration from 1 to 25 g/L was shown to induce *C. meneghiniana* size reduction and decreased relative pigment content, but these effects became observable in 30 months of cell growth in high-salinity conditions.^74^ Transfer to high-salinity conditions was also shown to affect the fluorescence characteristics of a marine diatom *Nitzschia* sp., which, however, required more than 1 year of acclimation to high salinities.^75^ It is assumed that extracellular polymeric substances, produced by diatoms and composed mostly of polysaccharides, proteins, lipids, etc.,^76^ prevent high-salinity shock.^77^ In the current study, the time that *C. meneghiniana* is mounted in high-salinity conditions is limited to 30 min of cooling to 200 K (Figure S1) or even less; therefore, staying in FCS should not significantly affect the cells.

At 270 K, the cell suspension is completely thawed, while at 230 K, cells are expected to be still in the frozen state. However, 230 K is close to the eutectic temperature of the water/NaCl system (251.8 K). Since ASP media can be considered as a diluted saline buffer (86.0 mM NaCl and 136.36 mM total molarity) containing cells, we suppose that FCS can exist below 251.8 K and probably reach 230 K or even lower. This more likely explains the slight increase in the fluorescence intensity and characteristic lifetimes observed at 230 K. However, these changes can also be associated with the onset of fluctuations between conformational substates of the protein matrix at around 230 K.^78^

In view of the above, we suppose that the measurements in the temperature range from 16 to 130 K are performed on cells frozen in an “intact” state and are therefore valuable for analyzing the function of the photosynthetic machinery at the whole-cell level, when it is considered as an interconnected chain rather than as separate components.

Let us consider the temperature dependence of the fluorescence spectral shapes and decay kinetics (Figures 3, 4, and Table S2). FCPs associated with PSII fluoresce at 675–680 nm.^16^ Due to efficient energy transfer to PSII, the antenna complexes should contribute to the spectra only at lower temperatures. The PSII core itself exhibits two fluorescence maxima: (i) 685 nm emission, originating from 683 nm states in CP47 and CP43 at 77 K, which blueshifts to 682 nm at RT, and (ii) 695 nm, commonly observed at 77 K and originating from red-absorbing CP47 chlorophyll, which is not visible at RT.^79^

In ref 79, it was demonstrated that at 4 K, the contribution of RC emission to the total emission of CP47-RC and PSII core complexes is extremely small, and the CP47-RC emission maximum is centered at 691 nm at 4 K. This coincides with the fluorescence peak position of *C. meneghiniana* cells measured at 16 K in the current study, indicating that the cellular-level spectra at 16 K are dominated by CP47 emission. Temperature increase to 40 K leads to the appearance of a 685 nm shoulder, whose contribution to total fluorescence appears equal to that of the 690 nm band (the bands are not spectrally resolved, but a blueshift from 689 nm occurs). This is followed by a significant decrease in the 690 nm band contribution at 77 K, which continues with further temperature increase to 130 and 180 K. This is consistent with excitation not becoming trapped at the CP47 chlorophyll responsible for 695 nm emission at higher temperatures. The energy gap between the 695 and 685 nm states (∼200 cm^−1^) is no longer much larger than the thermal energy (*k*_B_ *T* is approximately 90 cm^−1^ at 130 K). Considering the 685 nm fluorescence decay, considerably slowed-down kinetics at 16 K imply slower charge transfer in the RC^80^ as well as energy transfer within the PSII core. As the temperature gets higher, faster charge separation and energy transfer to the fluorescing states result in faster 685 nm fluorescence decay.

PSI-FCP fluorescence, when measured at 77 K, is characterized by the maximum position around 700 nm on a several picosecond time scale and around 720 nm at a nanosecond time scale and indicates the functional association of FCP to PSI and energy transfer between them in the membrane as shown with time-resolved fluorescence measurements.^55^ In stationary spectra at 77 K, the PSI-FCP emission from *C. meneghiniana* cells has a broad peak at ∼717 nm.^58^ Thus, the broad red band in our observed fluorescence spectra should contain contributions from PSI. Due to the fast decay of PSI fluorescence at RT, its influence on the spectra should be minimal. On the other hand, at lower temperatures, the intensity of the 720 nm band increases considerably, suggesting much larger contributions from PSI. Indeed, the temperature dependence of the intensity of this band is monotonous and does not resemble the somewhat visually more erratic behavior of the aggregates of the PSII-related FCP complexes, which exhibit a red-shifted component.^16^ Therefore, we can conclude that in the present case of *C. meneghiniana* cells, the increase of the red component intensity upon the lowering of the temperature is more likely dominated by the PSI emission. It follows that the longer decays measured at the 720 nm band reflect slower fluorescence decay in PSI than in PSII. Additionally, as the FCP antenna of PSII does not contribute more fluorescence at the red band at lower temperatures, it must transfer the energy to PSII cores very efficiently even at the lowest temperatures.

The intactness of the cells before starting measurements at cryogenic temperatures is essential for obtaining accurate data. There are dramatic differences between the spectra and fluorescence decays of almost completely dead cells (ASP/glycerol medium) and cells maintained in “intact” conditions prior to freezing (ASP only) at all temperatures (Figure 5).

We also note that measurements at 230 K, 270 K (Figure 3, C, D, G, H), and on thawed cells at RT (Figure S8) may provide additional information on their components in an uncoupled condition (i.e., swollen thylakoid membranes containing all components of the photosynthetic machinery), which could still retain their function. Indeed, the spectroscopic characteristics of dead cells can still be interpreted in terms of the components of the photosynthetic apparatus (Figure 9). If the dead-cell condition is not accounted for, this may lead to a completely erroneous description of the functioning of the photosynthetic machinery in living cells.

### 4.3 Inhibitor Analysis of the Photosynthetic Apparatus of *C. meneghiniana* Cells Investigated at Cryogenic Temperatures

Plastoquinones (PQs), along with the Cytochrome *b*_6_f complex and cytochrome c_6_ (which replaces plastocyanin in diatoms), form an intersystem electron chain that mediates the sequential functioning of both photosystems. The proton motive force, which consists of the transmembrane proton gradient and the transmembrane potential, is a major regulator of photosynthetic activity and photoprotection.^81^ By inhibition of electron flow or dissipation of the transmembrane proton gradient, the individual contributions of the components of the photosynthetic apparatus to the overall function of the system can be studied.

At RT, DCMU treatment leads to an increased fluorescence yield and longer characteristic lifetimes, as DCMU blocks electron transfer to Q_B_, resulting in a closed PSII-RC. FCCP, a proton uncoupler, also increases fluorescence yield and intensity-averaged characteristic lifetime when measured at RT, although the effect is less pronounced than in DCMU-treated cells. The ΔpH is crucial for nonphotochemical quenching, FCPa aggregation, and the conversion of diadinoxanthin (Ddx) to diatoxanthin (Dtx).^20^ Dissipation of the proton gradient likely inhibits the conversion of Ddx to Dtx, causing more energy to remain in the excited states of the chlorophylls, which differs from the effect of DCMU. However, at 16 K, the fluorescence spectra and decays of DCMU-treated, FCCP-treated, and untreated cells are very similar in both shape and numerical features, although DCMU- and FCCP-treated cells lack the fastest τ_1_ decay component. This similarity likely indicates that, at 16 K, fluorescence is dominated solely by CP47 of the PSII core: electron transfer is halted, and the PSII RC is closed, a state that occurs independently of the inhibitor’s action.

At higher temperatures (from 40 to 130 K), the broadening of the main fluorescence band due to the increased relative contribution of the 695 nm band occurs under both DCMU and FCCP treatment. In untreated cells, a sharp decrease in the red-absorbing CP47 chlorophyll contribution to the total PSII band fluorescence is observed. In contrast, DCMU and FCCP treatment leads to energy trapping by red-absorbing chlorophylls of CP47, which is observable up to 130 K. This trapping likely leads to the increased intensity average lifetimes observed in the 77–180 K spectral range (Figure 6), when the contribution of 695 nm states is comparable to that of 685 nm states, unlike in untreated cells where the 695 nm band is significantly suppressed (Figure 3). Interesting features are observed at 40 K in DCMU-treated cells compared to untreated cells: the spectrum is still dominated by 695 nm states, with an additional small red shift to 693 nm (from 691 nm at 16 K), and a faster fluorescence decay occurs compared to untreated and FCCP-treated cells. Such a decrease in τ_AVG_ is likely associated with this red shift to 693 nm, which places the maximum of the spectral band at the edge of the window used for fluorescence lifetime measurements (emission window 685 ± 10 nm). This artificially reduces its contribution to total fluorescence. Greater similarity between DCMU-treated and untreated cells in the 711 nm band behavior is expected, since it is very broad and also contains contributions from PSI, whose activity should not be affected by DCMU. Indeed, besides the constant offset, the behavior of τ_AVG_ at low temperatures is identical between untreated and DCMU-treated cells. The source of this constant increase in τ_AVG_ comes from PSII, not PSI.

The appearance of a shoulder at around 675 nm in the fluorescence spectra and its gradual increase as the temperature rises from 40 to 130 K are related to FCPs and occur in untreated cells as well as in cells treated with DCMU and FCCP.

Inhibitor analysis provides interesting data on the fluorescence of cells that underwent the freezing/thawing cycle, indicating that the photosynthetic machinery retains some functionality in damaged cells with swollen thylakoid membranes. The fluorescence characteristics of thawed DCMU-treated cells at 270 K do not resemble those of intact DCMU-treated cells but instead match those of thawed untreated cells (also at 270 K). The fluorescence decays are slower than those of intact cells but faster than those of intact DCMU-treated cells (Figure 6, S9 and S11). This indicates that closure of PSII is no longer dominating the signal, and that FCP disconnection from PSII and aggregation within the thylakoid membrane likely occurred, similar to untreated cells. However, FCCP treatment induces a significant decrease in τ_AVG_ during the final phases of thawing at 270 K, and this effect is even more pronounced for emission at 720 nm (Figure 6 and S12). This directly indicates that an additional mechanism responsible for the increased τ_AVG_ in untreated and DCMU-treated cells must exist, which can be inhibited by FCCP.

It was shown that DCMU does not affect diatoxanthin epoxidation in *C. meneghiniana* and that this diatom exhibits an alternative electron transport pathway through which NADPH can be oxidized, supplying the PQ pool with electrons.^82^ This indicates that even if electron transfer from PSII to the PQ pool is restricted by DCMU, electron transfer to PSI still occurs. We suggest that the increase in intensity and the slowing of fluorescence decays at 270 K occur due to closed PSII-RCs in some cells, and this effect is even enhanced by DCMU. However, alternative electron flow to PSI, corresponding NADPH consumption, and the formation of a proton gradient still take place. However, electron transfer is self-regulated to some extent, with the proton gradient acting as a rate-limiting factor for electron flow to PSI. This is reflected in the stabilization of τ_AVG_ and fluorescence intensity in the final phase of thawing. As a result, although the cells are damaged and a large portion may be dead, the FCPs and both photosystems can still respond to external stimuli, such as light irradiation.

FCCP treatment induces the complete destruction of this “fake” functional state of the photosynthetic apparatus. FCCP dissipates the proton gradient and removes the rate limitation of electron flow, resulting in uncontrolled electron sinking via PSI, which leads to its photodamage accompanied by quenching. The resulting significantly shorter τ_AVG_ and lower fluorescence intensity under FCCP support this hypothesis.

An additional important note is that the changes in τ_AVG_ induced by thawing are similar to the time-dependent changes in τ_AVG_ observed in cells after medium exchange to ASP/glycerol (Figure 2A,B), indicating that similar processes associated with cell damage and death occur in both cases. This means that measurements performed in the presence of glycerol, even if freezing was fast, are more likely to reflect not the intact cell but a “fake” functioning cell, in which some functions of the photosynthetic machinery can still be mimicked, but the overall operation is far from the real state observed in intact cells.

## 5 Conclusions

In this study, we establish that glycerol-free cryospectroscopy enables reliable investigation of photosynthetic processes in diatom cells frozen in an intact state, revealing native-state electronic excitation dynamics at the whole-cell level that is obscured in measurements performed in glycerol-containing cryo-media. Exposure of cells to glycerol causes rapid and severe changes, leading to immediate damage in more than a third of the cell population and significant alterations in the photosynthetic machinery during thylakoid membrane swelling. Changes in the fluorescence decay likely indicate detachment of FCPs from PS cores, possibly followed by their aggregation. More importantly, components of the photosynthetic apparatus may still retain some functions, which can create the false impression that the cell has been frozen in a normal, intact state and lead to an incorrect interpretation of the observed phenomena.

Avoiding glycerol allows investigation of the photosynthetic machinery in cells frozen in an “intact” state, which, in future, should lead to significant progress in understanding photosynthetic processes. Although the present work represents one of the initial steps in cryo-spectroscopy of intact photosynthetic machinery at the whole-cell level, it already reveals several characteristic spectroscopic features of electronic excitation dynamics, such as the dominance of CP47 emission at 16 K and its progressive quenching upon heating, the slowing of charge separation and energy transfer within PSII cores at cryogenic temperatures, and the likely major contribution of the PSI-FCP complex to the broad, far-red emission above 700 nm, with no resolvable contribution from the PSII antenna at the temporal resolution of the experimental approach used. However, a small antenna contribution cannot be excluded. Additionally, we believe our findings will encourage other researchers to select experimental conditions more carefully, avoiding the simple extrapolation of approaches used for simple structures to extremely complex systems.

The findings expand the understanding of the complexity and specificity of electronic excitation dynamics at the whole-cell level (rather than in isolated complexes) and are crucial not only for fundamental photosynthesis research. Applications span far beyond and can form the basis for plant engineering and design of new algal strains highly adaptive to changing environmental conditions and stress and thus contributing to sustainable development and biomass production.

## Supplementary Material



## Notes

Description of the fluorescence decay fitting technique and pairwise comparison *p*-values (PDF)
