# Supplementary material for: Whole-Cell Glycerol-free Fluorescence Cryo-Spectroscopy Reveals Electronic Excitation Dynamics in the Diatom Cyclotella meneghiniana
Source: JACS Au. 2026 Jul 8;6(9):5037–54. doi: 10.1021/jacsau.6c00630 (PMC13625515; doi:10.1021/jacsau.6c00630)
Supplement: Supplementary file 1 [file au6c00630_si_001.pdf]

Supporting Information

Whole-cell glycerol-free fluorescence cryo-spectroscopy reveals electronic excitation dynamics in the diatom *Cyclotella meneghiniana*.

Lena Golubewa[1,2]*, Yaraslau Padrez[2,3], Patricija Zemaityte[2,4], Danielis Rutkauskas[2], Claudia Büchel[5], and Andrius Gelzinis[1,2]*

[1] Institute of Chemical Physics, Faculty of Physics, Vilnius University, Sauletekio Av. 9, III bld., 10222, Vilnius, Lithuania

[2] Department of Molecular Compound Physics, State Research Institute Center for Physical Sciences and Technology, Sauletekio Av. 3, 10257, Vilnius, Lithuania

[3] Neuroscience Research Center, College of Medicine, Jiaxing University, Jiaxing, Zhejiang Province, 314001, China

[4] Life Science Center, Vilnius University, Sauletekio Av. 7, 10257, Vilnius, Lithuania

[5] Institute of Molecular Biosciences, Department of Biosciences, Goethe University Frankfurt, Max-von-Laue-Str. 9, 60438 Frankfurt, Germany

1. Supplementary Figures

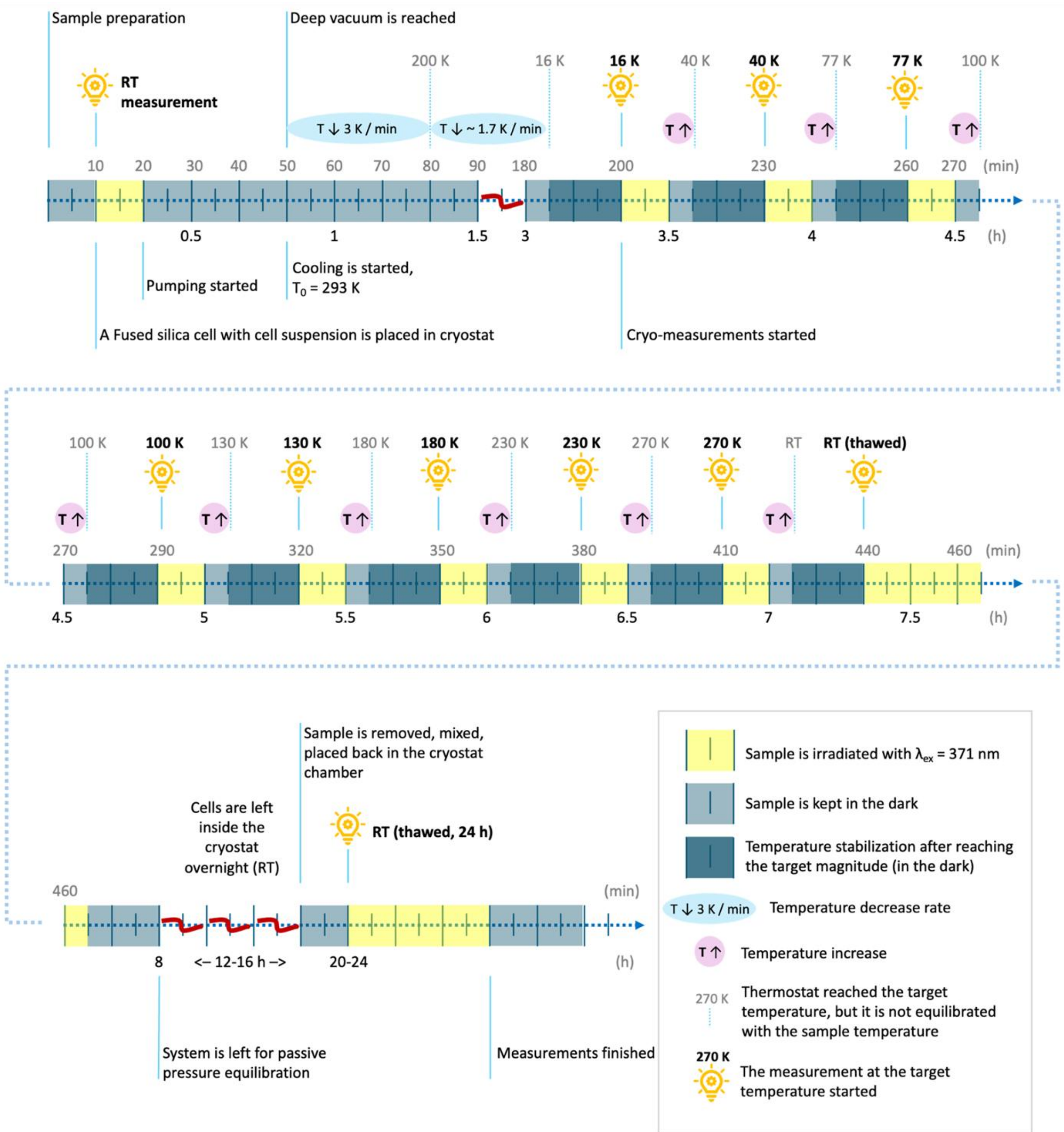

Figure S1. Timeline of the steady-state and time-resolved fluorescence measurements of cells *C. meneghiniana* at cryogenic temperatures.

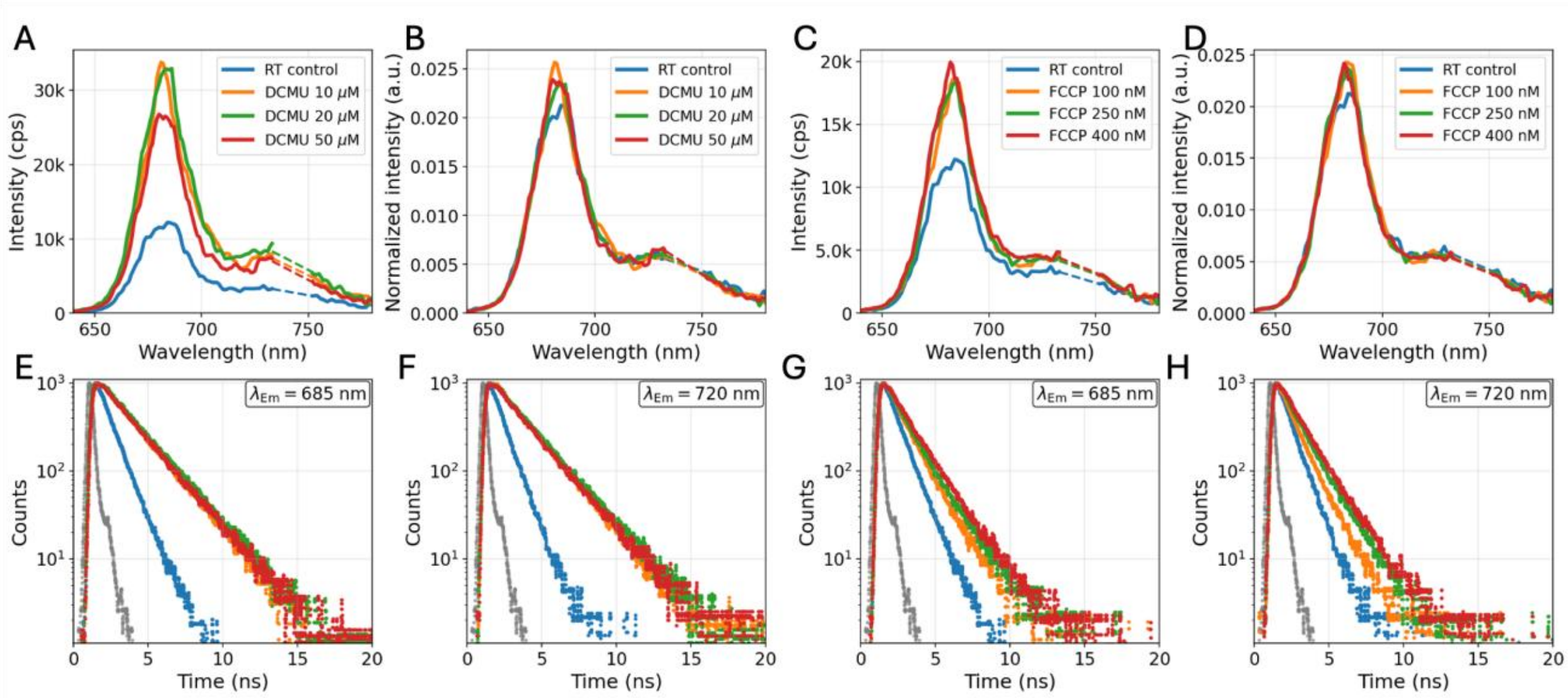

Figure S2. The dependence of the steady-state fluorescence spectra (A-D) and fluorescence decays (E-H) of cells *C. meneghiniana* on the concentration of DCMU (A, B, E, F) and FCCP (C, D, G, H) at different concentrations: A and C – emission spectra in absolute units, B and D – emission spectra normalized by area, E and G – fluorescence decays detected at 685 nm, F and H – fluorescence decays detected at 720 nm. The measurements were performed at RT at λ_{ex} = 371 nm. Prior measurements, cells were incubated with either DCMU or FCCP for 10 min and kept in the dark.

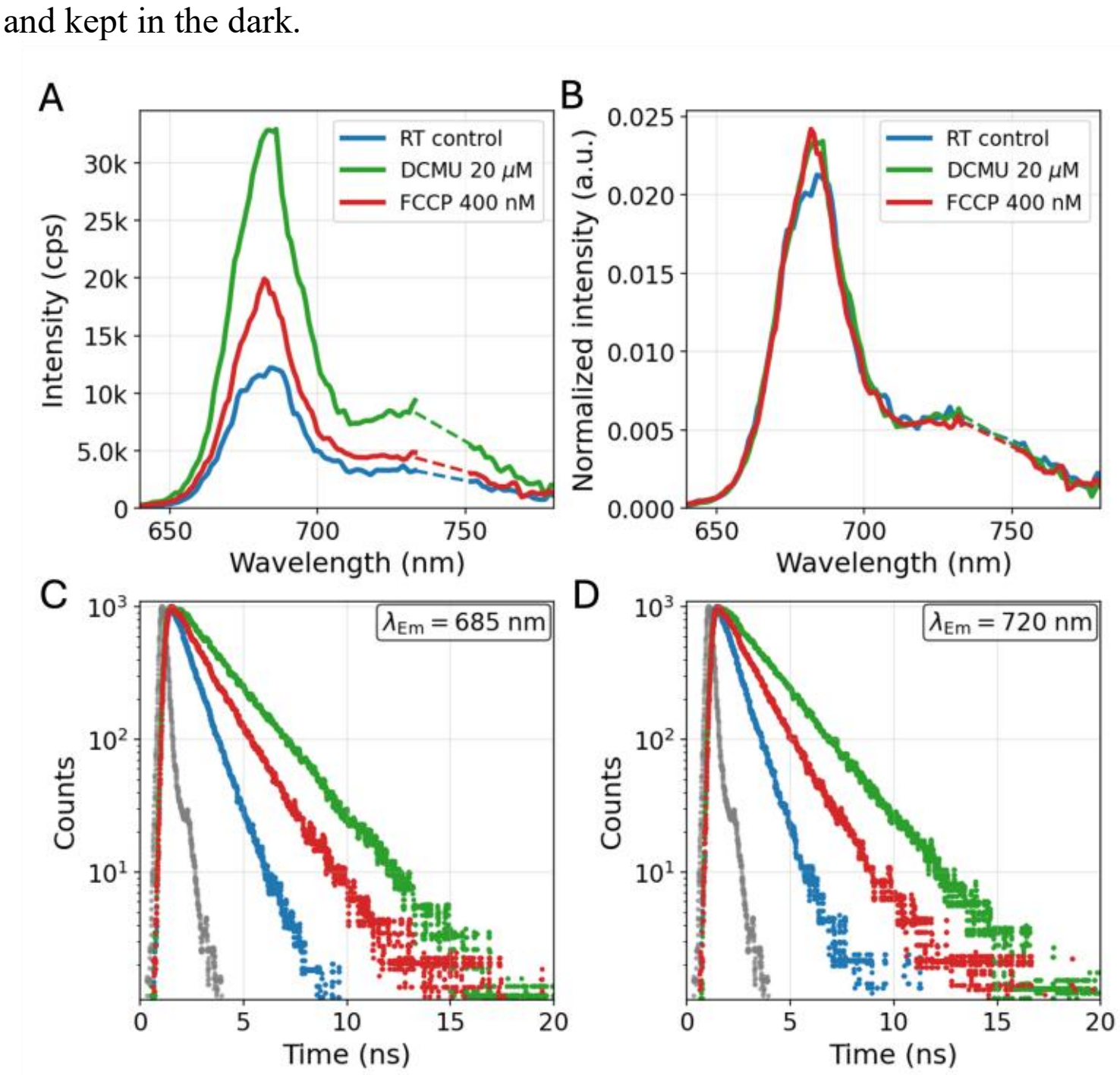

Figure S3. Comparison of steady-state fluorescence spectra (A-B) and fluorescence decay curves (C-D) of control cells and cells treated with 20 μM DCMU and 250 nM FCCP: A – emission spectra in absolute units, B – emission spectra normalized by area, C – fluorescence decays detected at 685 nm, D – fluorescence decays detected at 720 nm. The measurements were performed at RT at λ_{ex} = 371 nm. Prior measurements, cells were incubated with either DCMU or FCCP for 10 min in the dark.

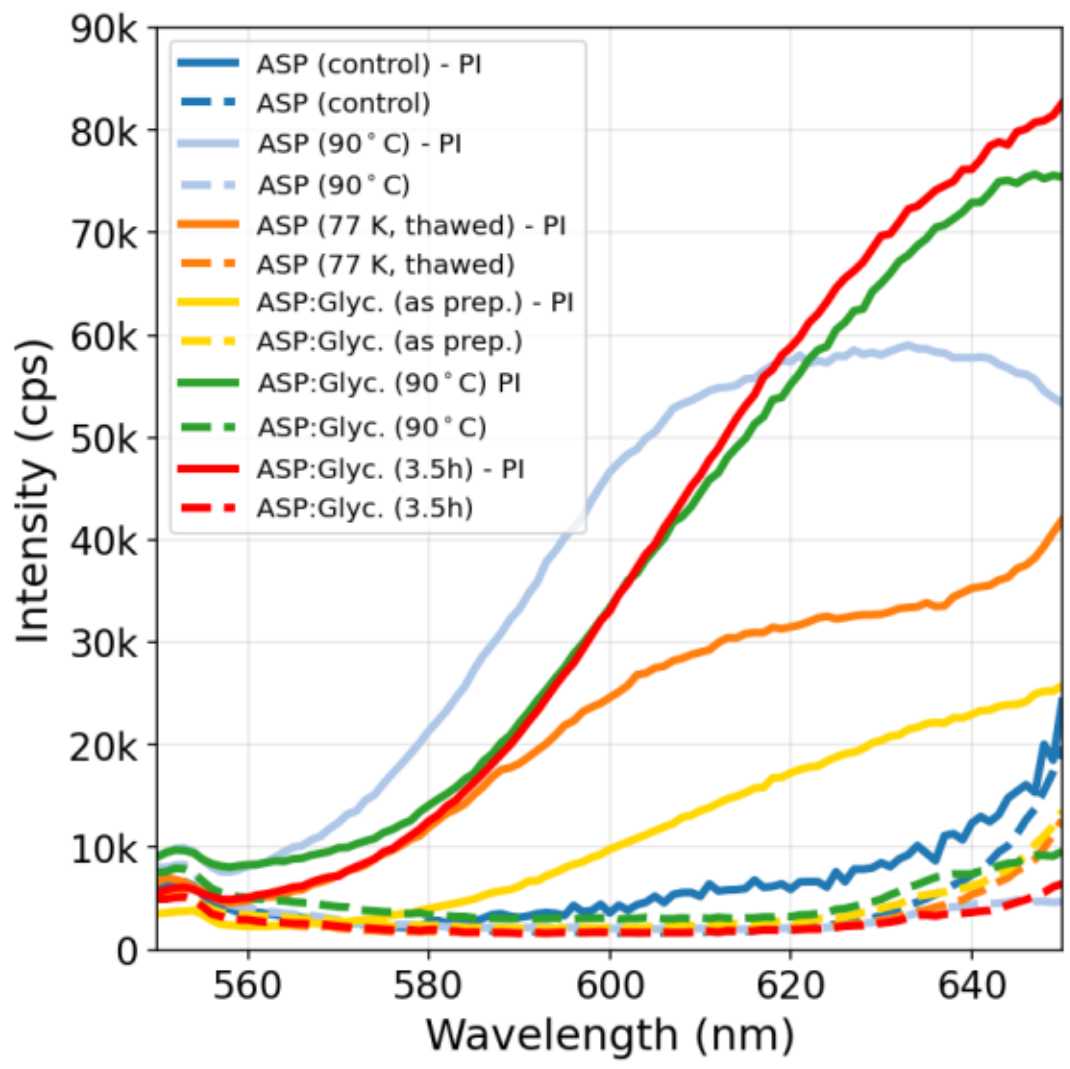

Figure S4. Propidium iodide (PI) spectra measured in *C. meneghiniana* cells and used for the calculation of the portion of the cells with compromised membrane integrity (**Figure 1F**). Solid line spectra correspond to cells incubated with PI for 30 min and dashed line spectra correspond to the spectra of cells without PI. Excitation wavelength was λ_{ex} = 535 nm.

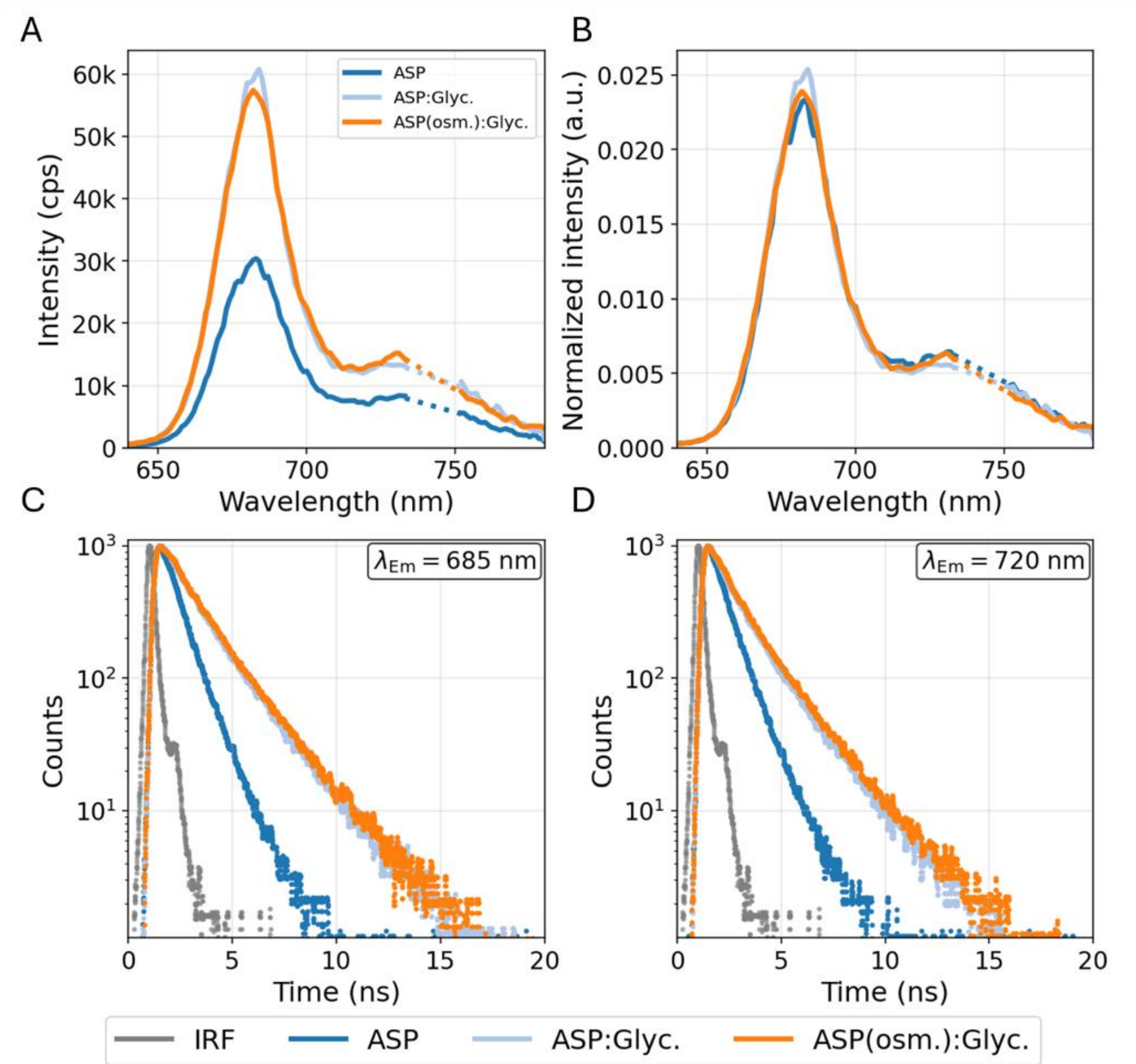

Figure S5. Salt concentration effect on the spectroscopic characteristics of *C. meneghiniana* in ASP:glycerol medium compared to ASP at RT. Panel A – the steady-state fluorescence spectra, B – normalized steady-state fluorescence spectra, C – time-resolved fluorescence decays at 685 nm, D – time-resolved fluorescence decays at 720 nm excitations of cells in ASP (blue line), ASP:glycerol (2:3, decreased salt concentration caused by ASP dilution with glycerol, referred to as ASP:Glyc.; light blue line) and ASP:glycerol (2:3, salt concentration adjusted to standard needed to maintain normal osmotic pressure, referred to as ASP(osm.):Glyc.; orange line). The spectra and decays were measured right after the media exchange. Dashed lines in A and B indicate regions with $2\lambda_{ex}$ (resulting from high scattering by the cell suspension and covering the 735-745 nm range) that were removed from the spectra to improve visualization and enable normalization of the spectra by area. Measurements were performed at RT at λ_{ex} = 371 nm.

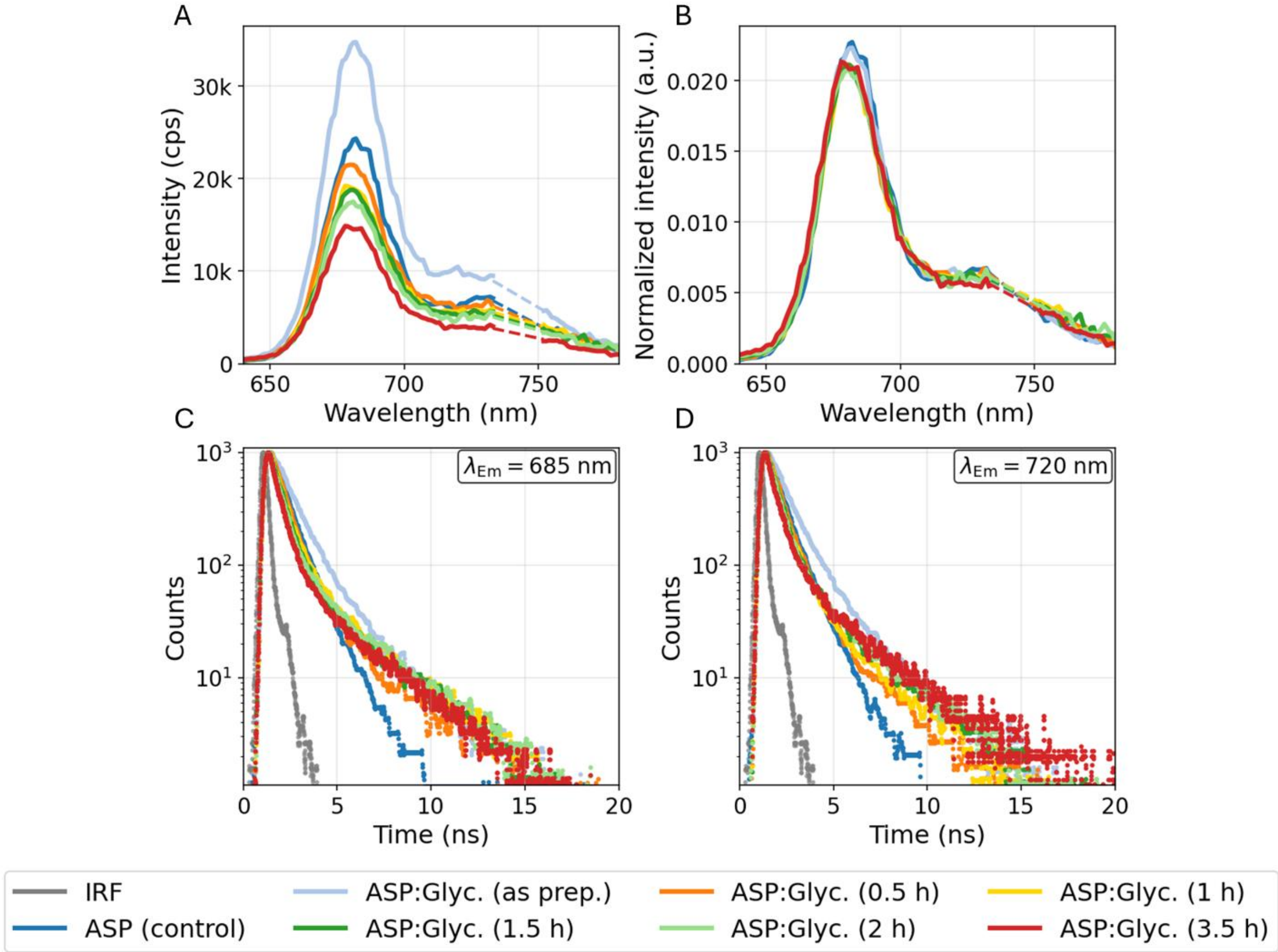

Figure S6. Temporal changes in steady-state fluorescence emission spectra and time-resolved fluorescence decays of cells *C. meneghiniana* measured in ASP media (control) and in media ASP:glycerol (2:3) (see **Figure 2**, A-B). Panel A – emission spectra in absolute units, B – emission spectra normalized by area, C – fluorescence decays detected at 685 nm, D – fluorescence decays detected at 720 nm. The measurements were performed at RT at $\lambda_{ex} = 371$ nm. Dashed lines in A and B indicate regions with $2\lambda_{ex}$ (resulting from high scattering by the cell suspension and covering the 735-745 nm range) that were removed from the spectra to improve visualization and enable normalization of the spectra by area.

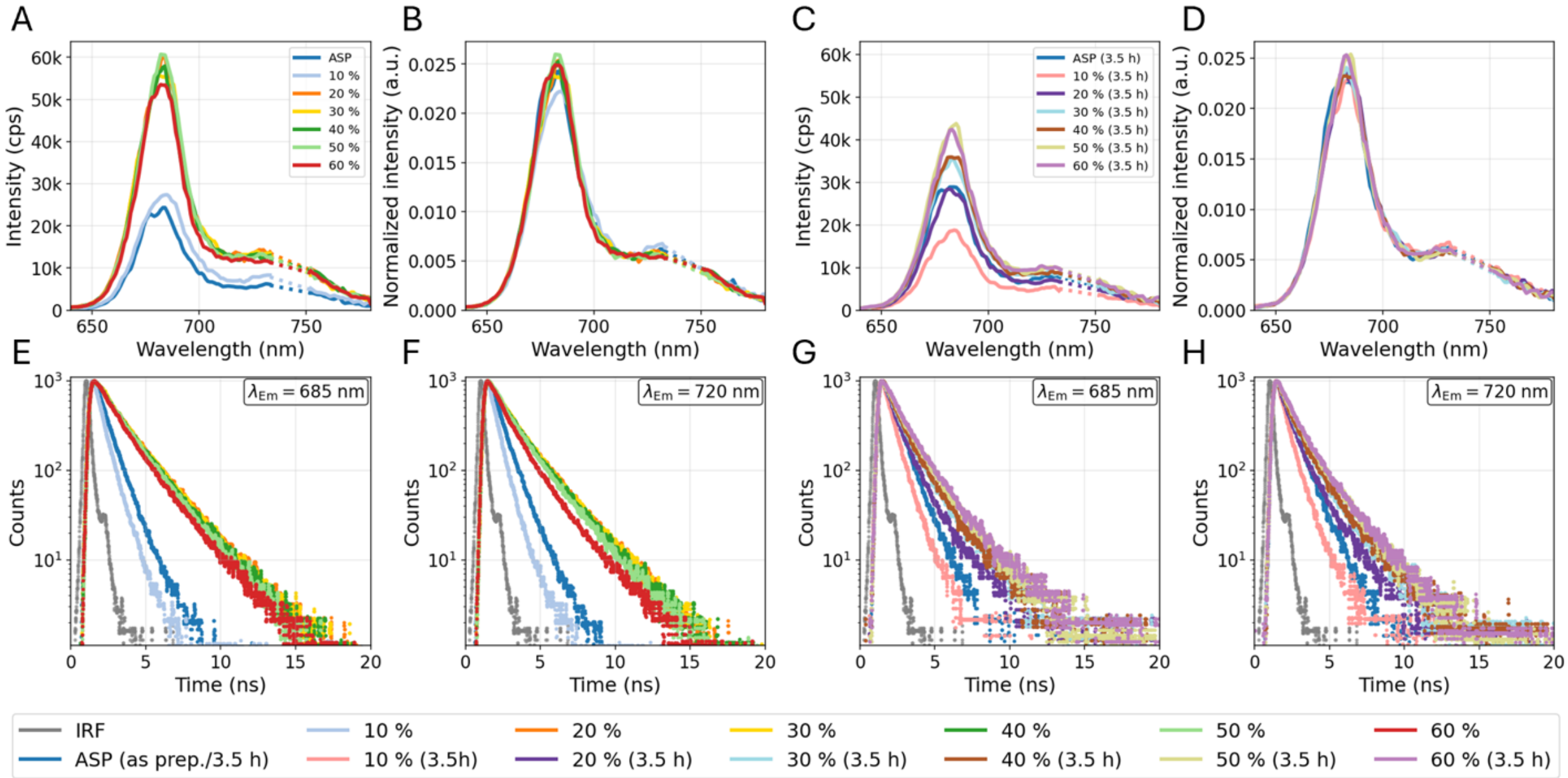

Figure S7. Influence of different glycerol concentrations on the steady-state fluorescence spectra (A-D) and fluorescence decay kinetics (E-H) of *C. meneghiniana* cells (**Figure 2, C-D**). Panel A – steady-state fluorescence spectra, B – spectra normalized by area for cells immediately after transfer to ASP:glycerol medium; C – steady-state fluorescence spectra and D – spectra normalized by area for cells after 3.5 h of transfer to ASP:glycerol medium; E – fluorescence decays measured at 685 nm and F – 720 nm immediately after cell transfer to ASP:glycerol medium; E – fluorescence decays measured at 685 nm and H – 720 nm after 3.5 h of cell transfer to ASP:glycerol medium. Glycerol content was varied from 0% to 60% in 10% increments. Excitation was at $\lambda_{ex} = 371$ nm. The number of cells in each measurement corresponded to the number of cells in the suspension with OD 0.1 measured in ASP. Measurements were performed at RT. Dashed lines in A and B indicate regions with $2\lambda_{ex}$ (resulting from high scattering by the cell suspension and covering the 735-745 nm range) that were removed from the spectra to improve visualization and enable normalization of the spectra by area.

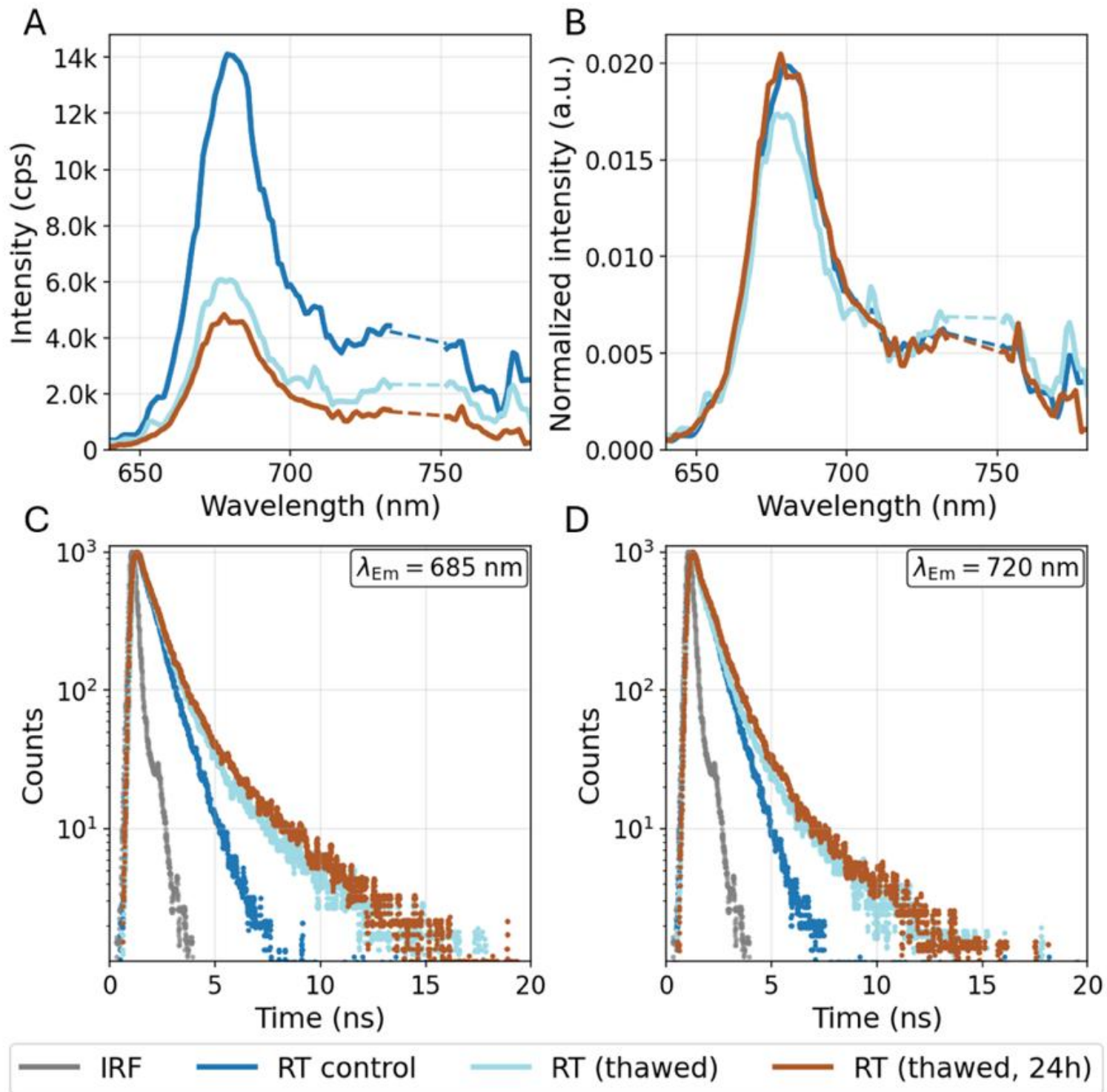

Figure S8. Steady-state fluorescence emission spectra and time-resolved fluorescence decays of *C. meneghiniana* cells measured in ASP medium at RT before and after the freezing/thawing cycle. Panel A shows emission spectra in absolute units; B shows emission spectra normalized by area; C shows fluorescence decays detected at 685 nm; D shows fluorescence decays detected at 720 nm. The dark blue curves represent measurements performed before the temperature decrease, referred to as 'control', and are provided in all panels for comparison. The emission slits for emission spectra and fluorescence decay measurements were 5 nm and 20 nm, respectively. Dashed lines in (A-B) indicate regions with $2\lambda_{ex}$ (resulting from high scattering by the cell suspension and covering the 735-745 nm range) that were removed from the spectra to improve visualization and enable normalization of the spectra by area. Small changes between two RT measurements of thawed samples with a 12-hour interval more likely originate from differences in the cell populations that are irradiated: in the RT (thawed) sample, the majority of the cells precipitated due to the long measurement time, and only significantly damaged floating cells are irradiated, while in the RT (thawed, 24 h) sample, all the cells (precipitated and floating) were mixed again. The differences between the decays measured before and after the freezing/thawing cycle demonstrate cell destruction after thawing (C-D).

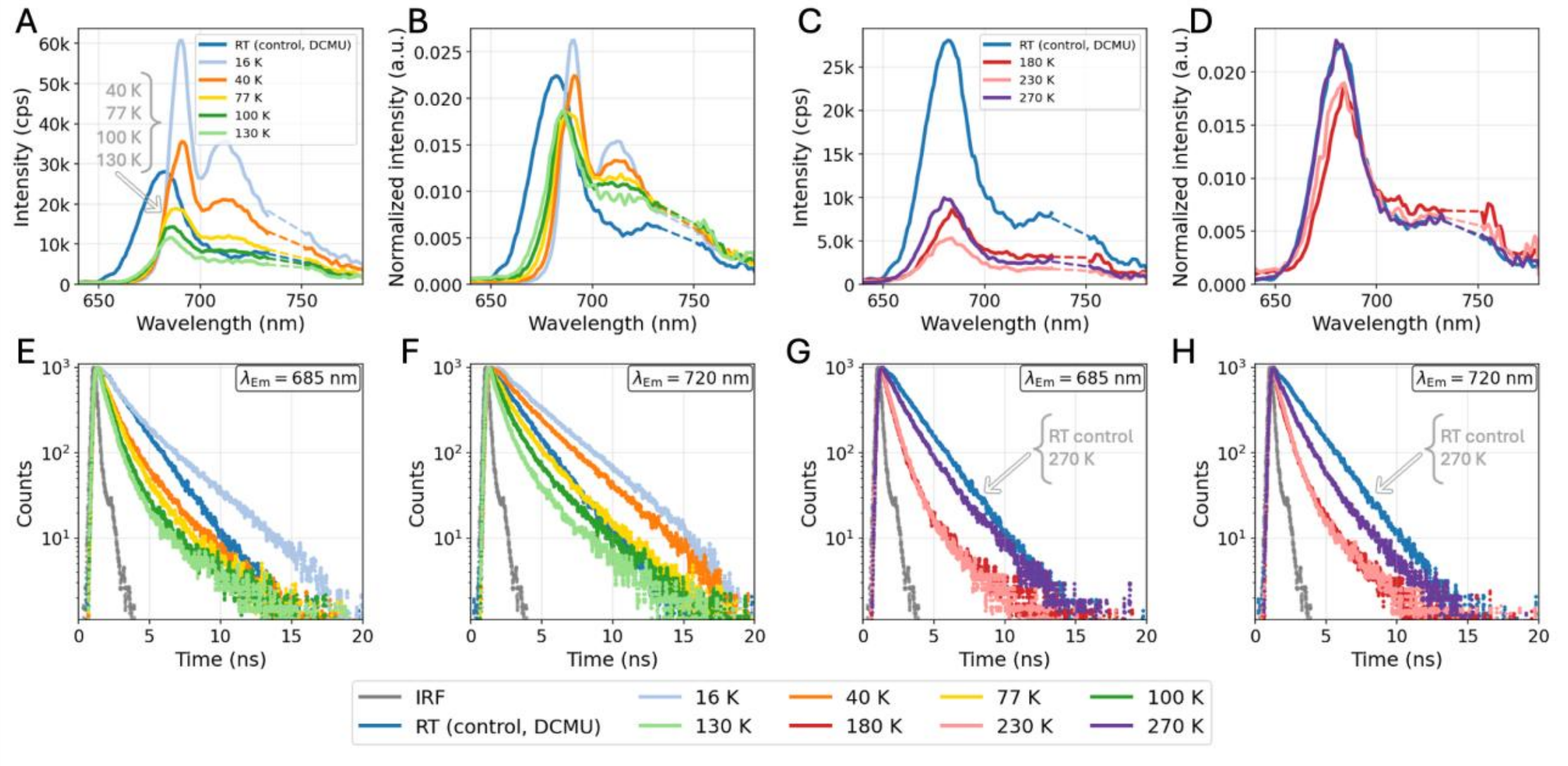

Figure S9. Temperature dependence of the steady-state fluorescence emission spectra (A–D) and time-resolved fluorescence decays (E–H) of *C. meneghiniana* cells measured in ASP medium under DCMU treatment: A and C show emission spectra in absolute units; B and D show emission spectra normalized by area; E and G show fluorescence decays detected at 685 nm; F and H show fluorescence decays detected at 720 nm (λ_{ex} = 371 nm). Panels A, B, E, and F correspond to the state of the cells considered 'intact', while C, D, G, and H correspond to the thawing/thawed state of the cells, considered the 'non-viable' state. The dark blue curves represent measurements performed before the temperature decrease, referred to as 'control', and are provided in all panels for comparison. The emission slits for emission spectra and fluorescence decay measurements were 5 nm and 20 nm, respectively. Dashed lines in A-D indicate regions with $2\lambda_{ex}$ (resulting from high scattering by the cell suspension and covering the 735–745 nm range) that were removed from the spectra to improve visualization and enable normalization of the spectra by area. Additional marks: A – gray-colored notation refers to the broadened bands, G and H – gray-colored notation points the similarities in decay curves measured at 270 K and at RT before freezing.

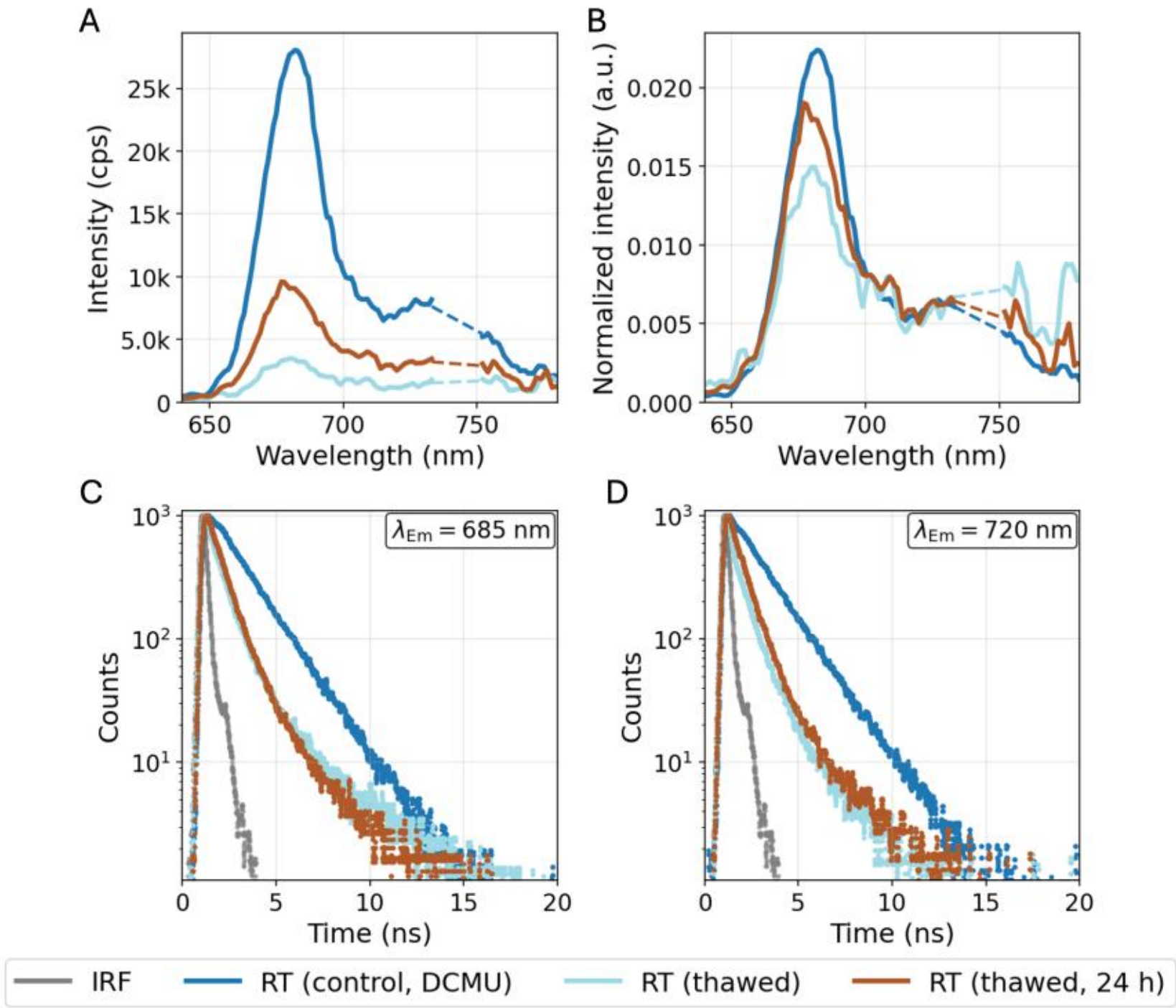

Figure S10. Steady-state fluorescence emission spectra (A–B) and time-resolved fluorescence decays (C–D) of *C. meneghiniana* cells measured in ASP medium at RT in the presence of 20 μM DCMU before and after the freezing/thawing cycle: A shows emission spectra in absolute units; B shows emission spectra normalized by area; C shows fluorescence decays detected at 685 nm; D show fluorescence decays detected at 720 nm (λ_{ex} = 371 nm). The dark blue curves represent measurements performed before the temperature decrease, referred to as 'control', and are provided in all panels for comparison. The emission slits for emission spectra and fluorescence decay measurements were 5 nm and 20 nm, respectively. Dashed lines in (A-B) indicate regions with $2\lambda_{ex}$ (resulting from high scattering by the cell suspension and covering the 735–745 nm range) that were removed from the spectra to improve visualization and enable normalization of the spectra by area.

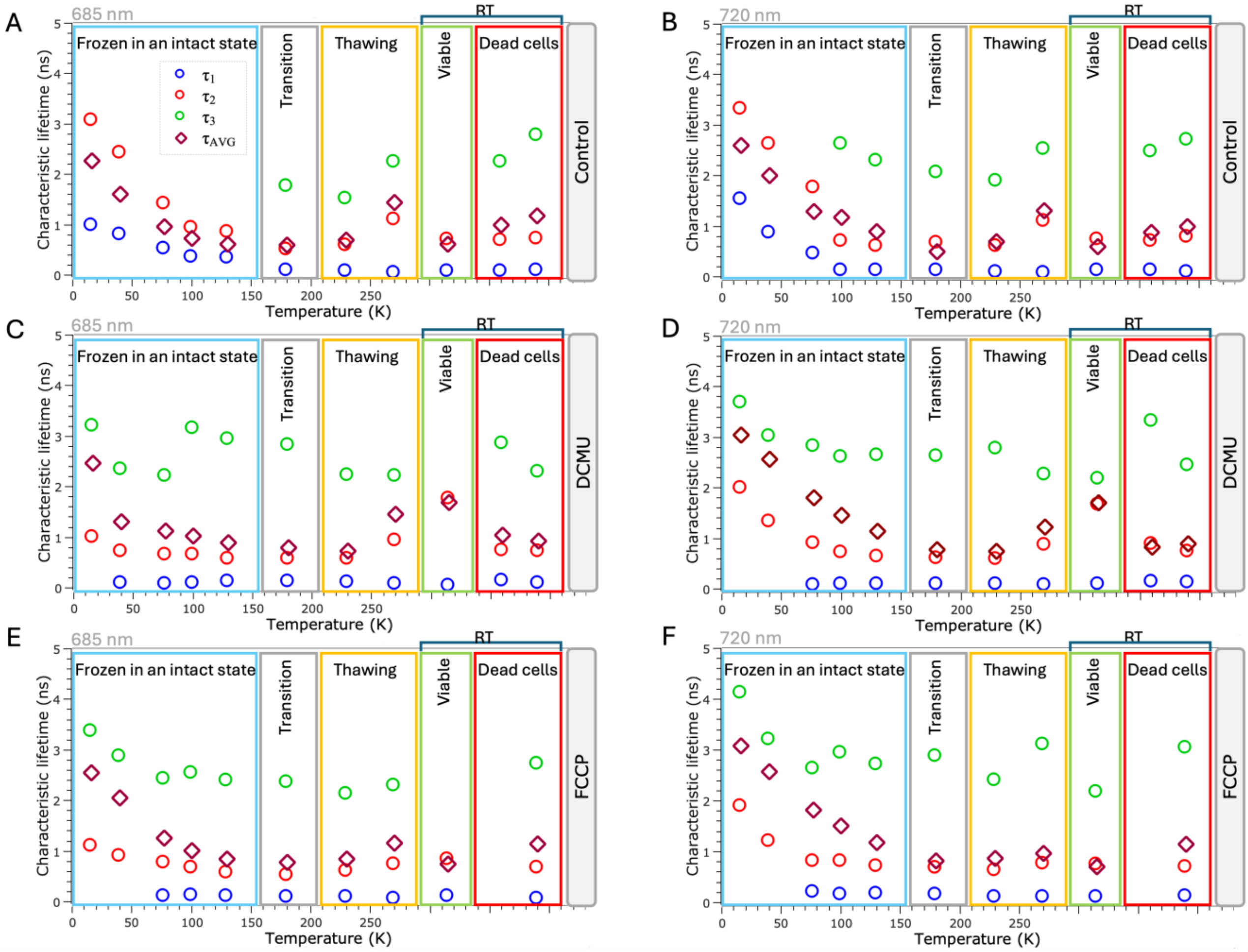

Figure S11. Temperature dependence of the characteristic lifetimes measured at 685 nm (A, C, E) and 720 nm (B, D, F) for untreated cells (A-B), 20 µM DCMU-treated cells (C-D) and 250 nM FCCP-treated cells (E-F). The cryogenic temperatures ranged from 16 K to 270 K. Green and red rectangles indicate room temperature measurements performed on the same sample before and after cryogenic measurements, respectively. The 'Thawed' label refers to the measurement performed on the same day as the measurements at other temperatures, while the '24 h' label indicates the measurement performed 24 hours after the measurements started. Five independent measurements were performed, and the data shown correspond to one representative measurement. The decays were fitted with either two or three exponential functions, depending on the fitting quality. The emission slit for the fluorescence decay measurements was 20 nm, λex = 371 nm. The legend in (a) corresponds to all panels.

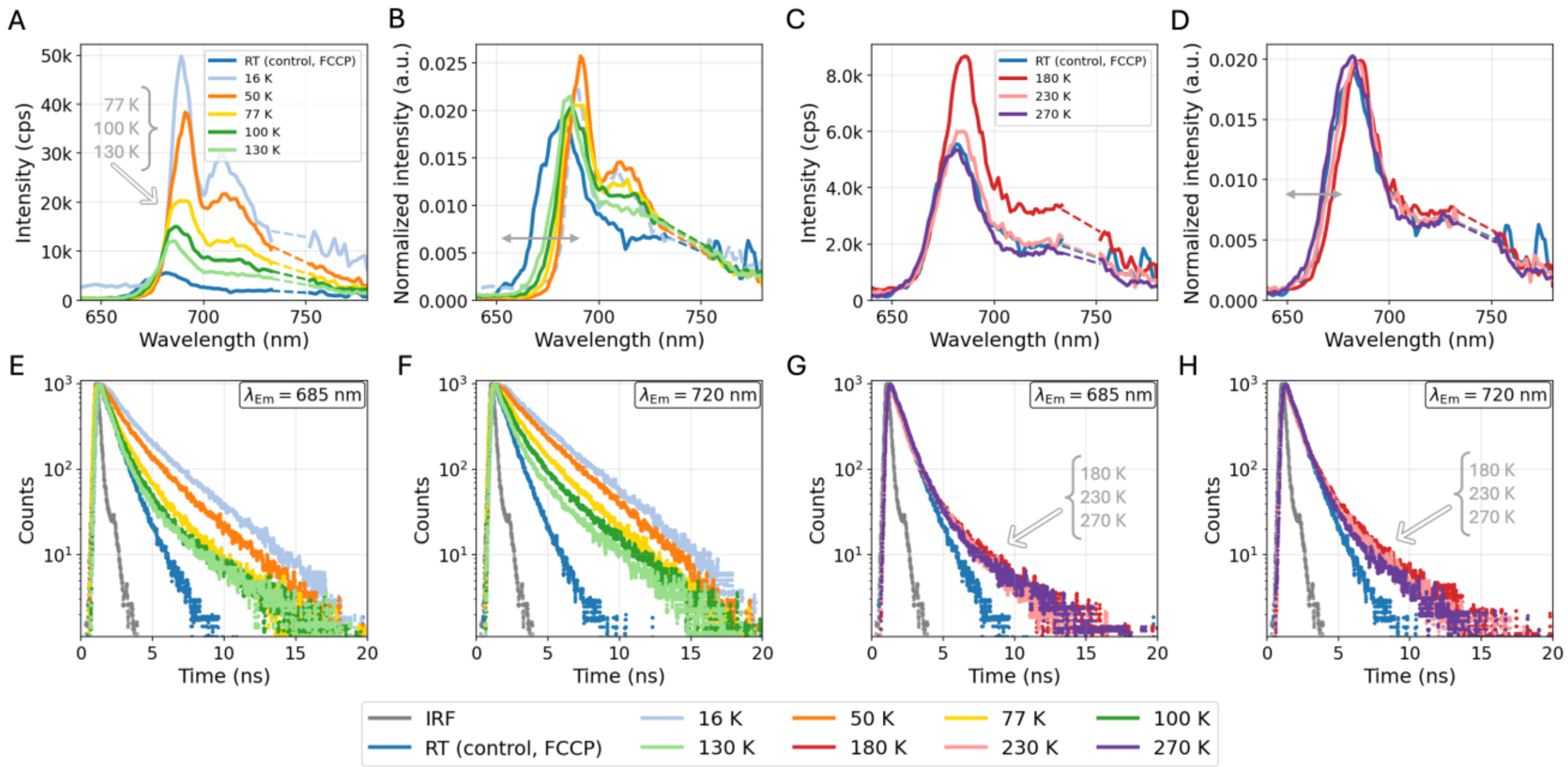

Figure S12. Temperature dependence of the steady-state fluorescence emission spectra (A–D) and time-resolved fluorescence decays (E–H) of *C. meneghiniana* cells treated with 250 nM FCCP for 10 min in the dark and measured in ASP medium: A and C show emission spectra in absolute units; B and D show emission spectra normalized by area; E and G show fluorescence decays detected at 685 nm; F and H show fluorescence decays detected at 720 nm (λ_{ex} = 371 nm). Panels A, B, E, and F correspond to the state of the cells considered 'intact', while C, D, G, and H correspond to the thawing/thawed state of the cells, considered the 'non-viable' state. The dark blue curves represent measurements performed before the temperature decrease, referred to as 'control', and are provided in all panels for comparison. The emission slits for emission spectra and fluorescence decay measurements were 5 nm and 20 nm, respectively. Dashed lines in (A-D) indicate regions with 2λ_{ex} (resulting from high scattering by the cell suspension and covering the 735–745 nm range) that were removed from the spectra to improve visualization and enable normalization of the spectra by area. Additional marks: A – gray-colored notation refers to the broadened bands, G and H – gray-colored notation points the similarities in decay curves measured at 180 K, 230 K and 270 K.

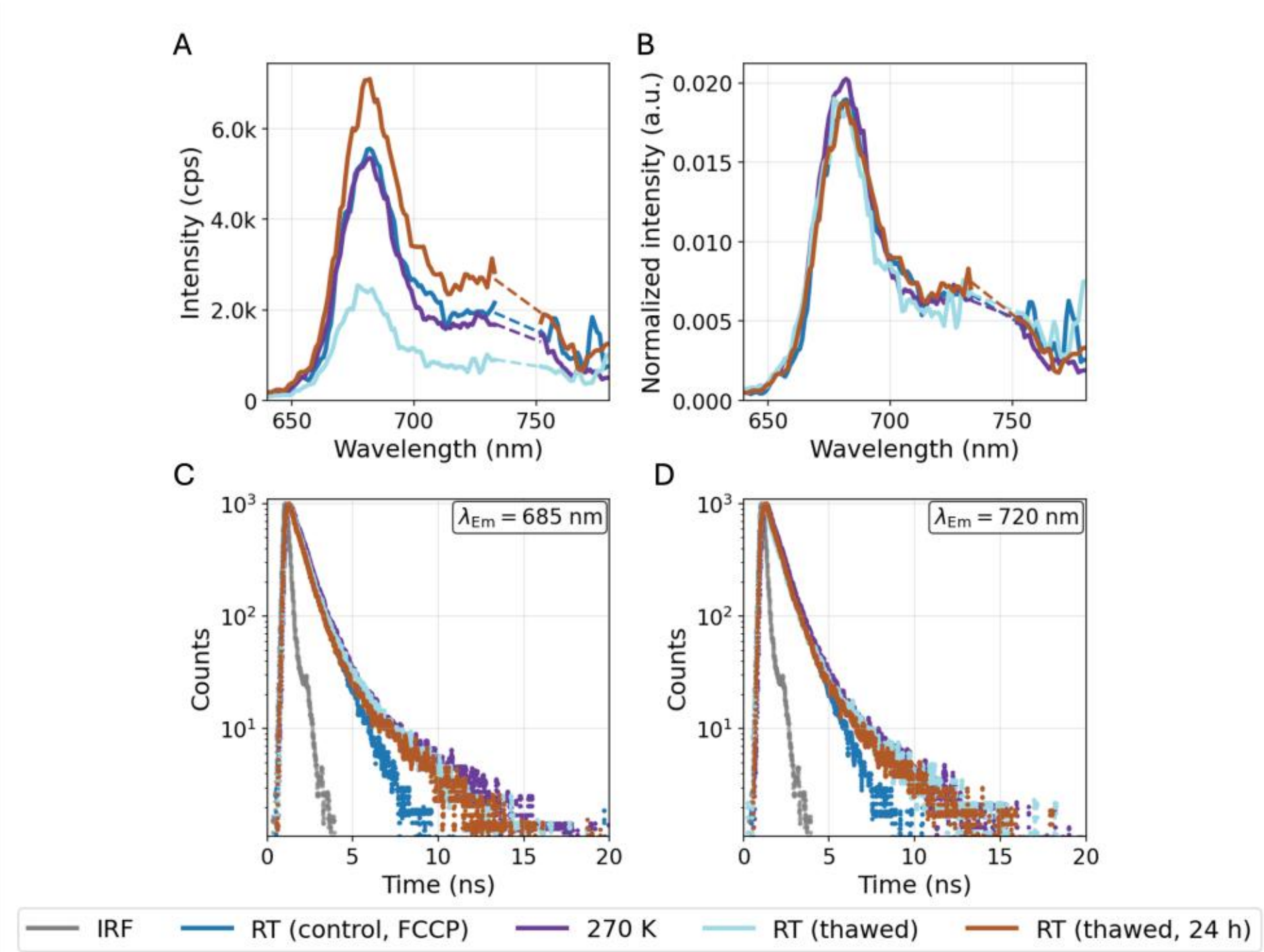

Figure S13. Steady-state fluorescence emission spectra (A–B) and time-resolved fluorescence decays (C–D) of *C. meneghiniana* cells measured in ASP medium at RT in the presence of 250 nM FCCP before and after the freezing/thawing cycle: A shows emission spectra in absolute units; B shows emission spectra normalized by area; C shows fluorescence decays detected at 685 nm; D show fluorescence decays detected at 720 nm (λ_{ex} = 371 nm). The dark blue curves represent measurements performed before the temperature decrease, referred to as 'control', and are provided in all panels for comparison. The emission slits for emission spectra and fluorescence decay measurements were 5 nm and 20 nm, respectively. Dashed lines in (A-B) indicate regions with $2\lambda_{ex}$ (resulting from high scattering by the cell suspension and covering the 735–745 nm range) that were removed from the spectra to improve visualization and enable normalization of the spectra by area.

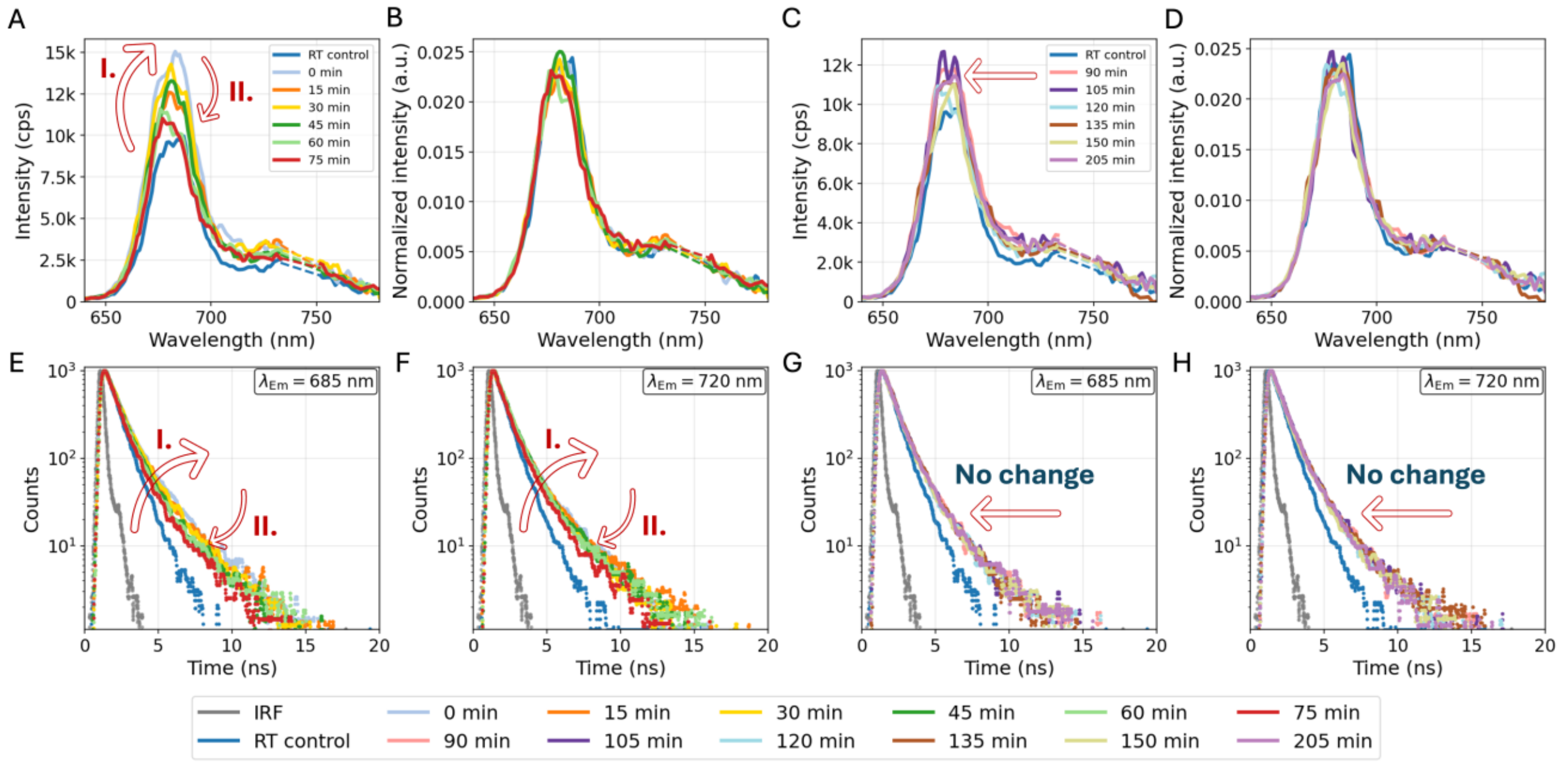

Figure S14. Steady-state fluorescence emission spectra (A-D) and time-resolved fluorescence decays (E-H) of cells *C. meneghiniana* measured before the freezing(at 77 K)-thawing cycle and over the time after thawing: A and C – emission spectra in absolute units, B and D – emission spectra normalized by area, E and G – fluorescence decays detected at 685 nm, F and H – fluorescence decays detected at 720 nm. Panel (A-B) and (E-F) correspond to the first half of the time after cell thawing, and (C-D) and (G-H) – to the second half. The measurements were performed at RT at λ_{ex} = 371 nm.

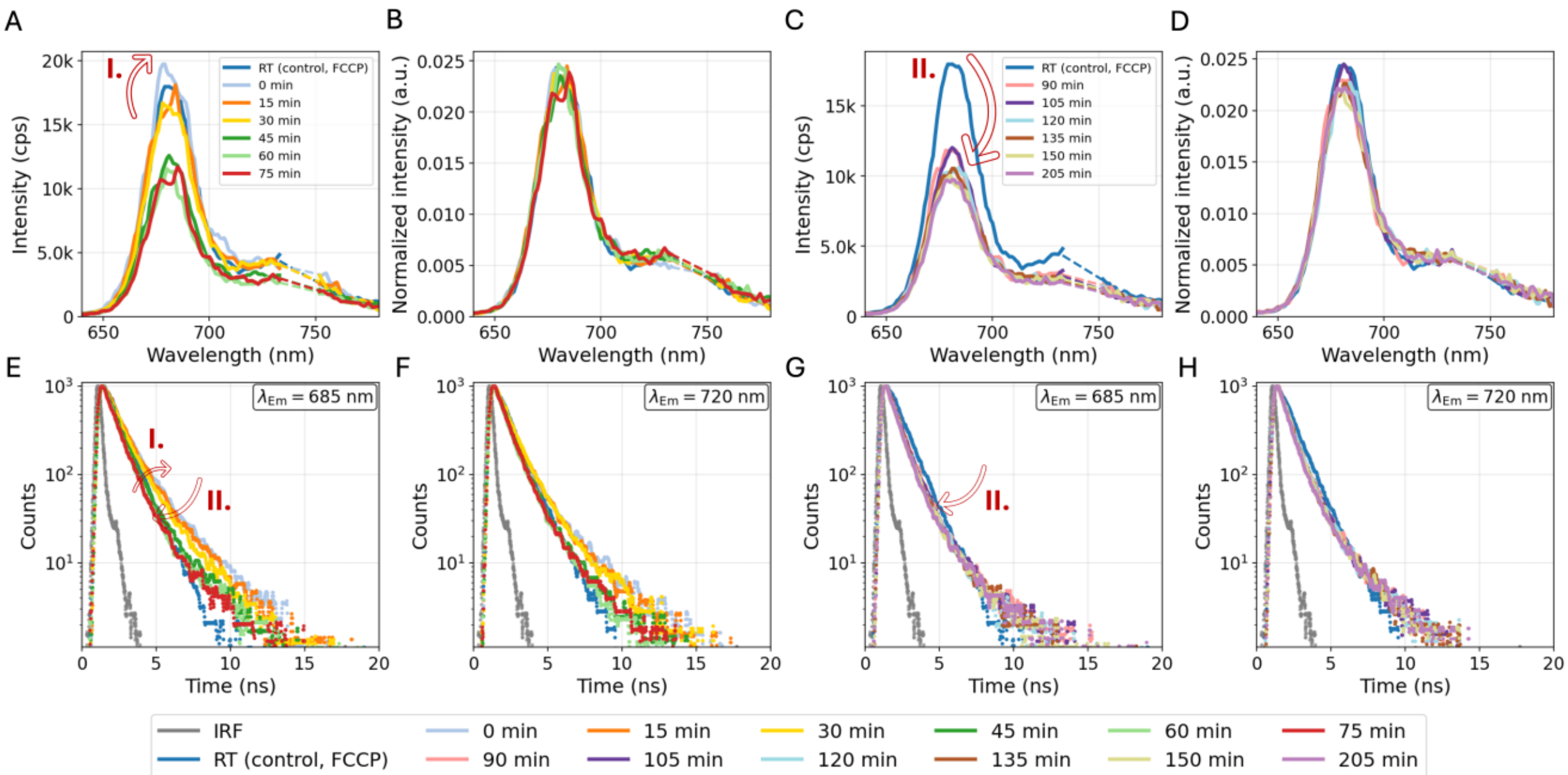

Figure S15. Steady-state fluorescence emission spectra (A-D) and time-resolved fluorescence decays (E-H) of cells *C. meneghiniana* treated with 250 nM FCCP and measured before the freezing(at 77K)-thawing cycle and over the time after thawing: A and C – emission spectra in absolute units, B and D – emission spectra normalized by area, E and G – fluorescence decays detected at 685 nm, F and H – fluorescence decays detected at 720 nm. Panel (A-B) and (E-F) correspond to the first half of the time after cell thawing, and (C-D) and (G-H) – to the second half. The measurements were performed at RT at λ_{ex} = 371 nm.

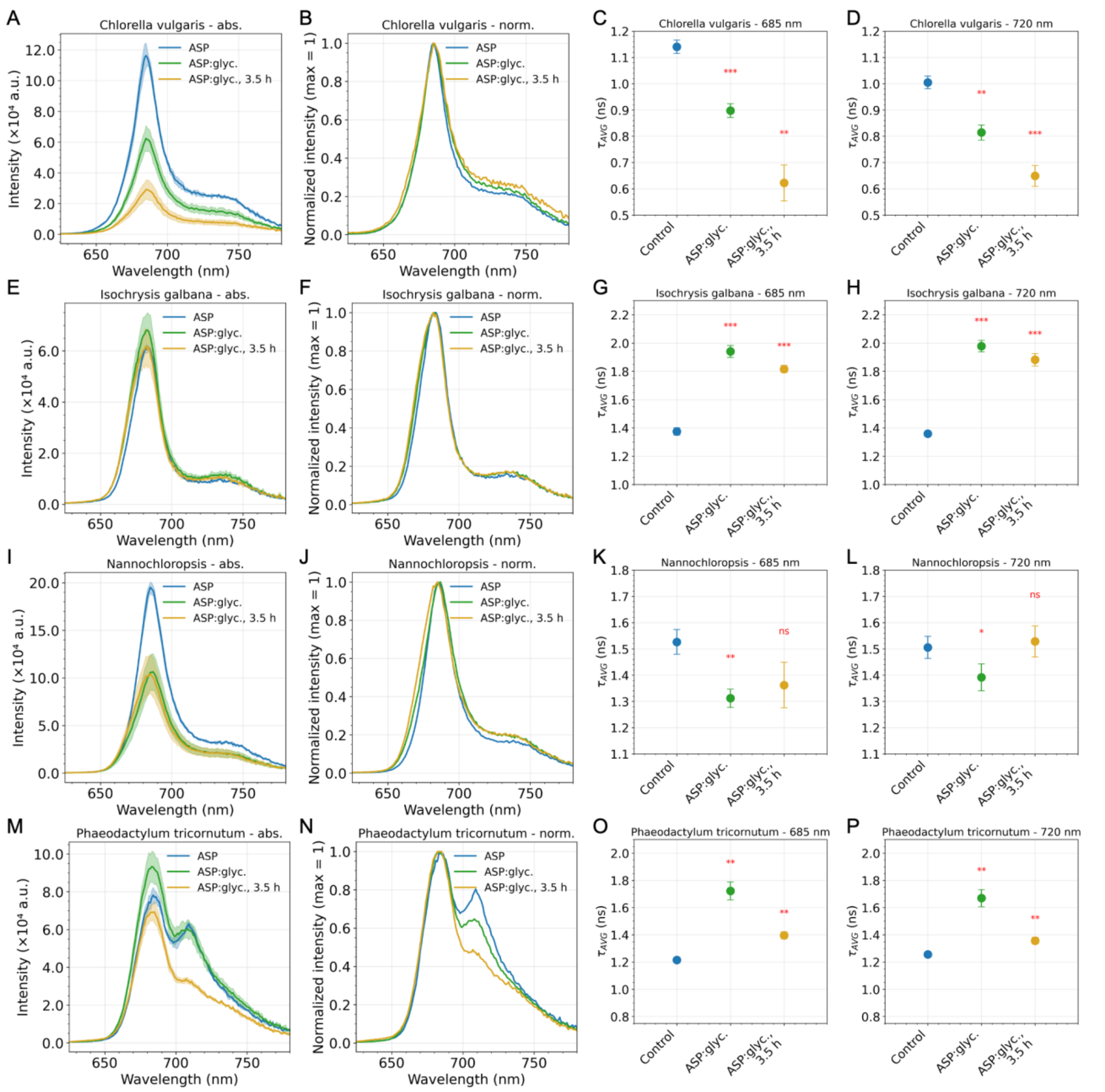

Figure S16. Effects of medium exchange from ASP to ASP:glycerol (2:3) on the spectroscopic characteristics of A–D: *C. vulgaris* (green alga), E–H: *I. galbana* (Haptophyte), I–L: *N. oceanica* (Eustigmatophyte), and M–P: *P. tricornutum* (pennate diatom) at RT. Results averaged from three independent measurements are shown. The first column shows averaged spectra with standard deviation in absolute intensity values; the second column shows fluorescence spectra normalized to maximum intensity; the third and fourth columns show intensity-averaged lifetimes calculated for decays measured at 685 nm and 720 nm emission, respectively. The excitation wavelength was λ_{ex} = 371 nm. Statistically significant differences between glycerol-related groups and control (ASP) are indicated as follows: $p < 0.05$*, $p < 0.01$**, $p < 0.001$***, ns – not statistically significant.

2. Supplementary Tables

Table S1. Characteristic lifetimes, corresponding pre-exponential factors (amplitudes) and fractional contributions of the characteristic lifetimes calculated for the time-resolved fluorescence decays measured at 685 nm and 720 nm of *C. meneghiniana* cells exposed to different media and conditions (numerical data corresponding to **Figure 1**). Characteristic lifetimes are presented in ns.

		**ASP control**	**ASP 90 °C**	**ASP 77 K (thawed)**	**Glycerol as prep.**	**Glycerol 90 °C**	**Glycerol 77 K (thawed)**	**Glycerol 3.5h**
685 nm	τ_1	0.17	0.15	0.13	0.13	0.16	0.18	0.2
	α_1	0.20	0.81	0.20	0.27	0.63	0.13	0.46
	f_1	0.05	0.33	0.03	0.05	0.16	0.04	0.2
	τ_2	0.72	0.69	0.68	0.65	0.63	0.56	0.50
	α_2	0.80	0.15	0.64	0.66	0.25	0.77	0.50
	f_2	0.95	0.28	0.57	0.65	0.25	0.66	0.53
	τ_3	-	3.40	1.96	2.50	3.32	2.06	2.88
	α_3	-	0.04	0.16	0.08	0.12	0.10	0.04
	f_3	-	0.39	0.39	0.30	0.59	0.31	0.28
	τ_{AVG}	**0.69**	**1.57**	**1.17**	**1.17**	**2.16**	**1.00**	**1.09**
	χ^2	1.30	1.41	1.51	1.46	1.52	1.46	1.41
720 nm	τ_1	0.16	0.18	0.13	0.21	0.14	0.17	0.20
	α_1	0.33	0.71	0.31	0.15	0.55	0.37	0.54
	f_1	0.09	0.31	0.06	0.04	0.12	0.11	0.22
	τ_2	0.76	0.64	0.67	0.68	0.62	0.62	0.54
	α_2	0.67	0.23	0.55	0.71	0.33	0.57	0.41
	f_2	0.91	0.35	0.54	0.57	0.31	0.62	0.47
	τ_3	-	2.92	1.97	2.31	3.10	2.32	2.88
	α_3	-	0.05	0.14	0.14	0.12	0.07	0.05
	f_3	-	0.35	0.40	0.39	0.58	0.27	0.31
	τ_{AVG}	**0.70**	**1.29**	**1.16**	**1.29**	**2.00**	**1.03**	**1.19**
	χ^2	1.38	1.10	0.99	1.49	1.53	0.95	0.98

Table S2. Characteristic lifetimes τ, corresponding pre-exponential factors *α* (amplitudes) and fractional contributions *f* of the characteristic lifetimes and average lifetimes τ_{AVG} calculated for the time-resolved fluorescence decays measured at 685 nm and 720 nm (numerical data correspond to **Figure 3**). Lifetimes are presented in ns. Measurements were performed in ASP medium (without glycerol).

		**16 K**	**40 K**	**77 K**	**100 K**	**130 K**	**180 K**	**230 K**	**270 K**	**RT (control)**	**RT (thawed)**	**RT (thawed, 24h)**
685 nm	τ_1	0.97	0.80	0.52	0.34	0.33	0.08	0.07	0.03	0.06	0.07	0.09
	α_1	0.66	0.75	0.73	0.60	0.68	0.54	0.61	0.76	0.63	0.64	0.52
	f_1	0.38	0.50	0.50	0.35	0.45	0.14	0.14	0.06	0.13	0.13	0.10
	τ_2	3.07	2.42	1.41	0.93	0.85	0.50	0.58	1.09	0.70	0.68	0.71
	α_2	0.34	0.25	0.27	0.40	0.32	0.44	0.35	0.18	0.37	0.33	0.43
	f_2	0.62	0.50	0.50	0.65	0.55	0.74	0.66	0.60	0.87	0.67	0.66
	τ_3	–	–	–	–	–	1.75	1.50	2.24	–	2.24	2.76
	α_3	–	–	–	–	–	0.02	0.04	0.05	–	0.03	0.04
	f_3	–	–	–	–	–	0.12	0.20	0.34	–	0.20	0.24
	τ_{AVG}	2.26	1.61	0.96	0.72	0.62	0.59	0.70	1.44	0.61	0.99	1.18
	χ^2	**1.43**	**1.39**	**1.39**	**1.47**	**1.14**	**1.28**	**1.40**	**1.48**	**1.45**	**1.28**	**1.45**
720 nm	τ_1	1.53	0.86	0.44	0.11	0.11	0.11	0.09	0.07	0.12	0.12	0.08
	α_1	0.59	0.62	0.68	0.58	0.61	0.66	0.64	0.70	0.59	0.69	0.67
	f_1	0.40	0.35	0.35	0.14	0.18	0.22	0.19	0.11	0.19	0.24	0.16
	τ_2	3.31	2.61	1.76	0.70	0.60	0.67	0.60	1.09	0.72	0.69	0.78
	α_2	0.41	0.38	0.32	0.37	0.35	0.32	0.34	0.26	0.41	0.28	0.30
	f_2	0.60	0.65	0.65	0.57	0.57	0.65	0.68	0.65	0.81	0.55	0.69
	τ_3	–	–	–	2.61	2.29	2.06	1.88	2.51	–	2.46	2.70
	α_3	–	–	–	0.05	0.04	0.02	0.02	0.04	–	0.03	0.02
	f_3	–	–	–	0.29	0.25	0.13	0.13	0.23	–	0.21	0.15
	τ_{AVG}	2.60	2.00	1.29	1.17	0.89	0.50	0.69	1.31	0.60	0.88	1.00
	χ^2	**1.33**	**1.22**	**1.48**	**1.18**	**1.15**	**1.19**	**1.12**	**1.40**	**1.30**	**1.15**	**1.36**

Table S3. Characteristic lifetimes, corresponding pre-exponential factors (amplitudes) and fractional contributions of the characteristic lifetimes calculated for the time-resolved fluorescence decays measured at 685 nm and 720 nm for the cells treated with 20 µM DCMU (numerical data correspond to **Figure S9** and **S10**). Characteristic lifetimes are presented in ns.

		**16 K**	**40 K**	**77 K**	**100 K**	**130 K**	**180 K**	**230 K**	**270 K**	**RT (DCMU, control)**	**RT (thawed)**	**RT (thawed, 24 h)**
685 nm	τ_1	–	0.09	0.06	0.09	0.11	0.11	0.10	0.06	0.03	0.13	0.09
	α_1	–	0.53	0.46	0.55	0.53	0.6	0.56	0.61	0.71	0.61	0.48
	f_1	–	0.09	0.06	0.12	0.15	0.2	0.17	0.07	0.04	0.18	0.10
	τ_2	0.99	0.71	0.64	0.65	0.57	0.57	0.57	0.92	1.76	0.73	0.71
	α_2	0.62	0.38	0.46	0.43	0.45	0.38	0.42	0.28	0.29	0.36	0.48
	f_2	0.33	0.51	0.61	0.70	0.68	0.66	0.69	0.46	0.96	0.61	0.73
	τ_3	3.20	2.33	2.20	3.14	2.93	2.81	2.22	2.20	–	2.85	2.29
	α_3	0.38	0.09	0.08	0.02	0.02	0.02	0.02	0.12	–	0.03	0.04
	f_3	0.67	0.41	0.34	0.18	0.17	0.15	0.15	0.47	–	0.20	0.18
	τ_{AVG}	**2.46**	**1.31**	**1.13**	**1.03**	**0.90**	**0.80**	**0.73**	**1.46**	**1.69**	**1.04**	**0.93**
	χ^2	**1.46**	**1.50**	**1.38**	**1.32**	**1.32**	**1.23**	**1.25**	**1.37**	**1.10**	**1.18**	**1.43**
720 nm	τ_1	–	–	0.07	0.08	0.08	0.10	0.09	0.06	0.08	0.14	0.12
	α_1	–	–	0.52	0.56	0.68	0.78	0.71	0.74	0.58	0.81	0.52
	f_1	–	–	0.05	0.08	0.16	0.32	0.24	0.13	0.06	0.38	0.14
	τ_2	1.99	1.23	0.90	0.71	0.63	0.60	0.58	0.86	1.65	0.88	0.72
	α_2	0.53	0.46	0.35	0.36	0.29	0.20	0.28	0.20	0.31	0.18	0.45
	f_2	0.38	0.26	0.45	0.49	0.54	0.52	0.62	0.52	0.65	0.53	0.71
	τ_3	3.67	3.02	2.81	2.60	2.64	2.61	2.76	2.25	2.17	3.31	2.44
	α_3	0.47	0.54	0.13	0.08	0.04	0.02	0.01	0.05	0.11	0.01	0.03
	f_3	0.62	0.74	0.50	0.42	0.30	0.17	0.14	0.35	0.29	0.09	0.15
	τ_{AVG}	**3.04**	**2.56**	**1.81**	**1.46**	**1.14**	**0.78**	**0.75**	**1.24**	**1.71**	**0.82**	**0.90**
	χ^2	**1.05**	**1.10**	**1.21**	**1.26**	**1.31**	**1.08**	**1.13**	**1.29**	**1.17**	**1.20**	**1.23**

Table S4. Characteristic lifetimes, corresponding pre-exponential factors (amplitudes) and fractional contributions of the characteristic lifetimes calculated for the time-resolved fluorescence decays measured at 685 nm and 720 nm for the cells treated with 250 nM FCCP (numerical data correspond to **Figure S12** and **S13**). Characteristic lifetimes are presented in ns.

		**16 K**	**40 K**	**77 K**	**100 K**	**130 K**	**180 K**	**230 K**	**270 K**	**RT (FCCP, control)**	**RT (thawed)**	**RT (thawed, 24 h)**
685 nm	τ_1	-	-	0.10	0.12	0.10	0.08	0.08	0.05	0.10	0.09	0.05
	α_1	-	-	0.38	0.40	0.43	0.53	0.56	0.69	0.53	0.51	0.66
	f_1	-	-	0.06	0.09	0.10	0.13	0.13	0.10	0.12	0.11	0.10
	τ_2	1.09	0.90	0.76	0.66	0.56	0.51	0.59	0.73	0.83	0.68	0.66
	α_2	0.63	0.69	0.53	0.56	0.53	0.45	0.40	0.26	0.48	0.46	0.31
	f_2	0.36	0.41	0.62	0.70	0.72	0.70	0.66	0.58	0.88	0.68	0.63
	τ_3	3.37	2.87	2.41	2.54	2.38	2.35	2.12	2.29	-	2.51	2.71
	α_3	0.37	0.31	0.09	0.04	0.03	0.02	0.04	0.05	-	0.04	0.03
	f_3	0.64	0.59	0.32	0.21	0.18	0.17	0.21	0.32	-	0.21	0.27
	τ_{AVG}	**2.55**	**2.06**	**1.25**	**1.01**	**0.85**	**0.77**	**0.84**	**1.16**	**0.74**	**1.01**	**1.16**
	χ^2	**1.26**	**1.40**	**1.46**	**1.44**	**1.37**	**1.25**	**1.28**	**1.22**	**1.44**	**1.35**	**1.44**
720 nm	τ_1	-	-	0.20	0.14	0.16	0.14	0.10	0.10	0.09	0.10	0.11
	α_1	-	-	0.22	0.52	0.62	0.74	0.66	0.76	0.77	0.61	0.53
	f_1	-	-	0.04	0.12	0.21	0.34	0.21	0.25	0.26	0.17	0.12
	τ_2	1.89	1.19	0.81	0.80	0.71	0.67	0.62	0.76	0.73	0.68	0.69
	α_2	0.65	0.54	0.54	0.40	0.33	0.24	0.31	0.23	0.22	0.36	0.43
	f_2	0.47	0.31	0.53	0.53	0.53	0.53	0.53	0.53	0.53	0.53	0.53
	τ_3	4.12	3.19	2.63	2.94	2.71	2.88	2.39	3.09	2.16	2.68	3.04
	α_3	0.35	0.46	0.24	0.08	0.05	0.02	0.03	0.02	0.01	0.03	0.04
	f_3	0.53	0.69	0.09	0.09	0.09	0.09	0.09	0.09	0.09	0.09	0.09
	τ_{AVG}	**3.08**	**2.58**	**1.82**	**1.51**	**1.18**	**0.82**	**0.87**	**0.97**	**0.70**	**0.97**	**1.15**
	χ^2	**1.08**	**1.43**	**1.36**	**1.30**	**1.31**	**1.28**	**1.28**	**1.32**	**1.43**	**1.05**	**1.28**

Table S5. Characteristic lifetimes, corresponding pre-exponential factors (amplitudes) and fractional contributions of the characteristic lifetimes calculated for the time-resolved fluorescence decays measured at 720 nm for the cells exposed to ASP:glycerol (2:3) at temperatures 16 K, 40 K, 77 K, 100 K, 130 K, 180 K and RT (measured before cooling). Numerical data correspond to **Figure 5**. Characteristic lifetimes are presented in ns.

		**16 K**	**40 K**	**77 K**	**100 K**	**130 K**	**180 K**	**RT (control)**
720 nm	τ_1	0.79	0.56	0.78	0.05	0.09	0.11	0.79
	α_1	0.00	0.00	0.51	0.55	0.42	0.42	0.00
	f_1	0.00*	0.00*	0.22	0.04	0.05	0.09	0.00*
	τ_2	1.88	1.44	1.78	0.82	0.70	0.58	1.88
	α_2	0.44	0.48	0.16	0.31	0.45	0.51	0.44
	f_2	0.27	0.27	0.16	0.36	0.44	0.59	0.27
	τ_3	3.91	3.65	3.27	3.04	2.77	2.34	3.91
	α_3	0.56	0.52	0.33	0.14	0.13	0.07	0.56
	f_3	0.73	0.73	0.61	0.61	0.50	0.32	0.73
	τ_{AVG}	**3.35**	**3.06**	**2.47**	**2.14**	**1.71**	**1.10**	**3.35**
	χ^2	2.14	1.73	1.48	1.41	1.29	1.27	2.14

** indicates fractional contributions < 1%*

3. Description of the fluorescence decay fitting technique

The detailed algorithm of the experimental fluorescence decays fitting with the lifefit.tcspc Python module is described below.

Algorithm description:
Experimental fluorescence decays are represented as a sum of n exponential components convolved with IRF and added background (constant):

$$y_{\mathrm{model}}(t) = \sum_{i=1}^{n} A_i \cdot \left(\mathrm{IRF}_{\mathrm{shifted}}(t) * e^{-t/\tau_i}\right) + B,$$

where A_i are the amplitudes of the ith component (pre-exponential factors; ≥ 0), τ_i are the ith fluorescence lifetimes (≥ 0), B is a background (constant, ≥ 0), $*$ refers to the convolution operator, $\mathrm{IRF}_{\mathrm{shifted}}$ is the experimental IRF shifted by Δ channels (with sub-channel precision).
The convolution is performed using Fast Fourier Transform (FFT, numpy.fft). The experimental data are considered as Poisson-distributed counts with the following weights, used in the outer least-squares fit (step 1 below):

$$\omega_j = 1 \Big/ \sqrt{y_{exp}(t_j) + 1},$$

where $y_{exp}(t_j)$ are the intensity counts, const 1 prevents division by 0.
The fitting involves two nested levels of optimization. An outer loop, which uses the Trust Region Reflective (TRF) algorithm (default selection by scipy.optimize.curve_fit because the parameter bounds are used) [S1, S2], adjusts the lifetimes τ_i and the instrument response shift Δ, while an inner loop solves a NNLS problem to find the best amplitudes and background for each candidate set (i.e., (τ_i, Δ) proposed by outer optimization algorithm), together minimizing the weighted difference between the measured decay and the model.
Algorithmic steps:
1. Data preparation:

- (i) The read-out fluorescence decays and experimental IRF were changed as an array of channel numbers and intensity counts. In our experiments, initial data had 4048 channels with 0.004883 ns per channel.
- (ii) The time axis was shifted so that the time t = 0 corresponded to the maximum of the decays.
- (iii) Poisson weights were computed.

2. Fitting initialization

- (i) The first guesses for $\tau_i^{(0)}$ were provided. They were: tau0 = [1], tau0 = [0.2, 3], tau0 = [0.2, 1, 3] and tau0 = [0.2, 1, 3, 10] for one-, two-, three- and four-exponential fitting and the IRF shift $\Delta^{(0)}$ was set to 0 channels (by default).
- (ii) The boundaries for τI and Δ were left as default: $\tau_i \in (0, \infty)$, $\Delta \in (-\infty, \infty)$.

3. Outer iteration (TRF algorithm, scipy.optimize.curve_fit)
A parameter vector p = $(\Delta, \tau_1, \ldots, \tau_n)$ was used as an input in the outer iteration and processed as follows:

(a) IRF shifting: The experimental IRF was shifted by Δ channels using Enderlein method [S3]:

$$\mathrm{IRF}_{\mathrm{shifted}}(t) = (1 - \delta) \cdot \mathrm{IRF}(t - \lfloor\Delta\rfloor) + \delta \cdot \mathrm{IRF}(t - \lceil\Delta\rceil),$$

where $\delta = \Delta - \lfloor\Delta\rfloor$, which enabled sub-pixel shifts ($\lfloor\Delta\rfloor$ refers to the greatest integer $\leq \Delta$; $\lceil\Delta\rceil$ – the smallest integer $\geq \Delta$).

(b) Generation of the convolved exponentials: For each lifetime τ_i, a single-exponential decay e^{-t/τ_i} was created on the same time grid as the IRF, and then the convolution with the shifted IRF was computed using FFT for speed:

$$C_i(t) = \mathrm{IRF}_{\mathrm{shifted}}(t) * e^{-t/\tau_i} = \mathcal{F}^{-1}\left(\mathcal{F}[\mathrm{IRF}_{\mathrm{shifted}}] \cdot \mathcal{F}\left[e^{-t/\tau_i}\right]\right).$$

(c) Then, the matrix $A = [C_1(t), C_2(t), \ldots, C_n(t), 1]$ of a dimension $m \times (n+1)$ (rows = time channels, columns = n exponentials + background), was built:

$$A = \begin{bmatrix} C_1(t_1) & C_2(t_1) & \cdots & C_n(t_1) & 1 \\ C_1(t_2) & C_2(t_2) & \cdots & C_n(t_2) & 1 \\ \vdots & \vdots & \ddots & \vdots & \vdots \\ C_1(t_m) & C_2(t_m) & \cdots & C_n(t_m) & 1 \end{bmatrix}$$

(d) The non-negative least squares problem was solved for amplitudes and background:

$$\min_{\mathrm{x}\geq 0} \left\| A\mathrm{x} - \mathrm{y}_{\mathrm{exp}} \right\|_2^2,$$

where $\mathrm{x} = (A_1, \ldots, A_n, B)^T$ and $\mathrm{y}_{\mathrm{exp}}$ is a vector of experimental counts. The scipy.optimize.nnls, which uses the active-set method of Lawson and Hansom [S4, S5], was used. It was iteratively found which components should be set to zero. Then, a constrained least-squares problem was solved for the remaining positive components. The convergence criteria were as follows: (i) the Karush-Kuhn-Tucker conditions were properly satisfied, or (ii) maximum iterations ($\max_{\mathrm{iter}} = 3 \times (n+1)$) were reached. If $\max_{\mathrm{iter}}$ was exceeded, then a 'RuntimeError' was raised.

(e) Considering $y_{fit} = A\mathrm{x}$ as a fitted decay, the weighted residual sum of squares was computed:

$$\chi^2(\mathrm{p}_k) = \sum_{j=1}^{m} \omega_j \left(y_{exp}(t_j) - (A\mathrm{x})_j\right)^2,$$

where ω_j are the Poisson weights. Then, the χ^2 value was returned to the TRF optimizer.

(f) The parameters were updated. Based on χ^2, a new set of lifetimes and a shift were computed. The iterations were repeated until convergence.

The default values for convergence for the outer loop were used:

- Gradient – gtol = 1×10^{-8}
- Parameter change – xtol = 1×10^{-8}
- Function change – ftol = 1×10^{-8}
- Max evaluations – max_nfev = 100 × (n + 1) (i.e., for 3-exponential, stop after 400 evaluations).

4. Results output

As an output, the following data was exported:

- Optimal lifetimes τ_i and std (from the diagonal of the covariance matrix computed by curve_fit).
- Optimal amplitudes from the final NNLS solution
- Background B
- IRF shift Δ (in ns, $\Delta \cdot t_{step}$) with std
- Fitted decay curves and residuals, χ^2.

5. Estimation of the appropriate number of exponentials

The number of exponentials was selected based on the χ^2 value (fit was considered acceptable if $\chi^2 \leq$ 1.5). Additionally, weighted residuals plots were analyzed (weighted residuals were centered around

zero, no clear waves or trends were observed, approx. 95 % of points are within ± 2). An example of the 1-, 2-, 3- and 4-exponential fits, corresponding χ^2 and weighted residual plots are shown in **Figure S17**.

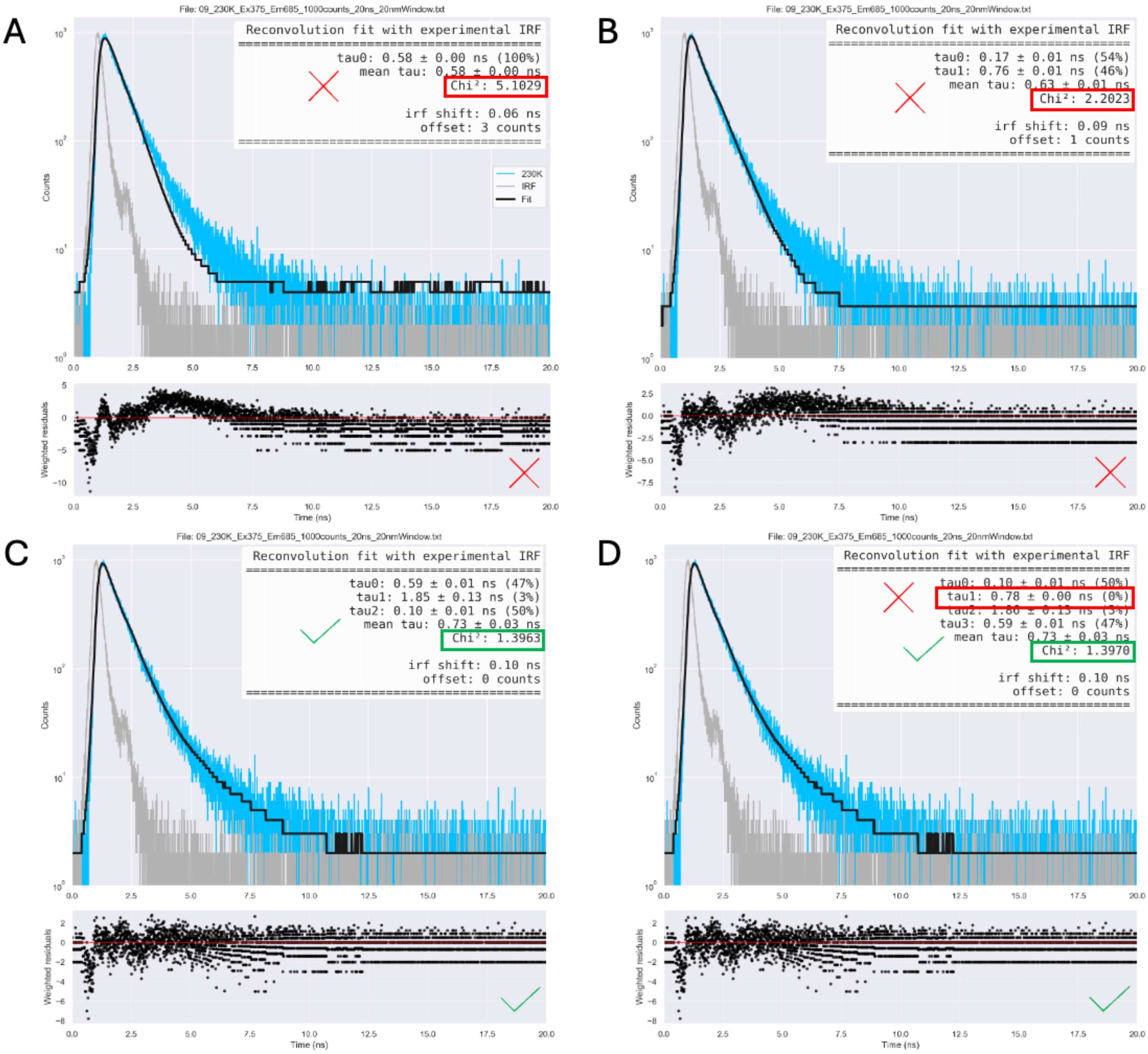

Figure S17. The results of fitting of fluorescence decay with (A) one-, (B) two-, (C) three- and (D) four-exponentials. Three-exponential fit (C) is selected as an appropriate one: χ^2<1.5, >95% of points are within ± 2 (weighted residuals). Four-exponential fit (D) does not improve χ^2, additional exponential comes with 0% contribution to the total decay function.

References:

[S1] Coleman, T. F.; Li, Y. A Reflective Newton Method for Minimizing a Quadratic Function Subject to Bounds on Some of the Variables. SIAM Journal on Optimization 1996, 6 (4), 1040–1058. https://doi.org/10.1137/S1052623494240456.
[S2] SciPy documentation: scipy.optimize.curve_fit – SciPy v1.17.0 Manual. https://docs.scipy.org/doc/scipy/reference/generated/scipy.optimize.curve_fit.html (accessed 2026-05-26).
[S3] Enderlein, J.; Erdmann, R. Fast Fitting of Multi-Exponential Decay Curves. Opt. Commun. 1997, 134 (1–6), 371–378. https://doi.org/10.1016/S0030-4018(96)00384-7.

[S4] Lawson, C. L.; Hanson, R. J. Solving Least Squares Problems; Society for Industrial and Applied Mathematics, 1995. https://doi.org/10.1137/1.9781611971217.
[S5] SciPy documentation: scipy.optimize.nnls (Lawson-Hanson) NNLS – SciPy v1.17.0 Manual. https://docs.scipy.org/doc/scipy/reference/generated/scipy.optimize.nnls.html (accessed 2026-05-26).

4. Pairwise Comparison *p*-Values

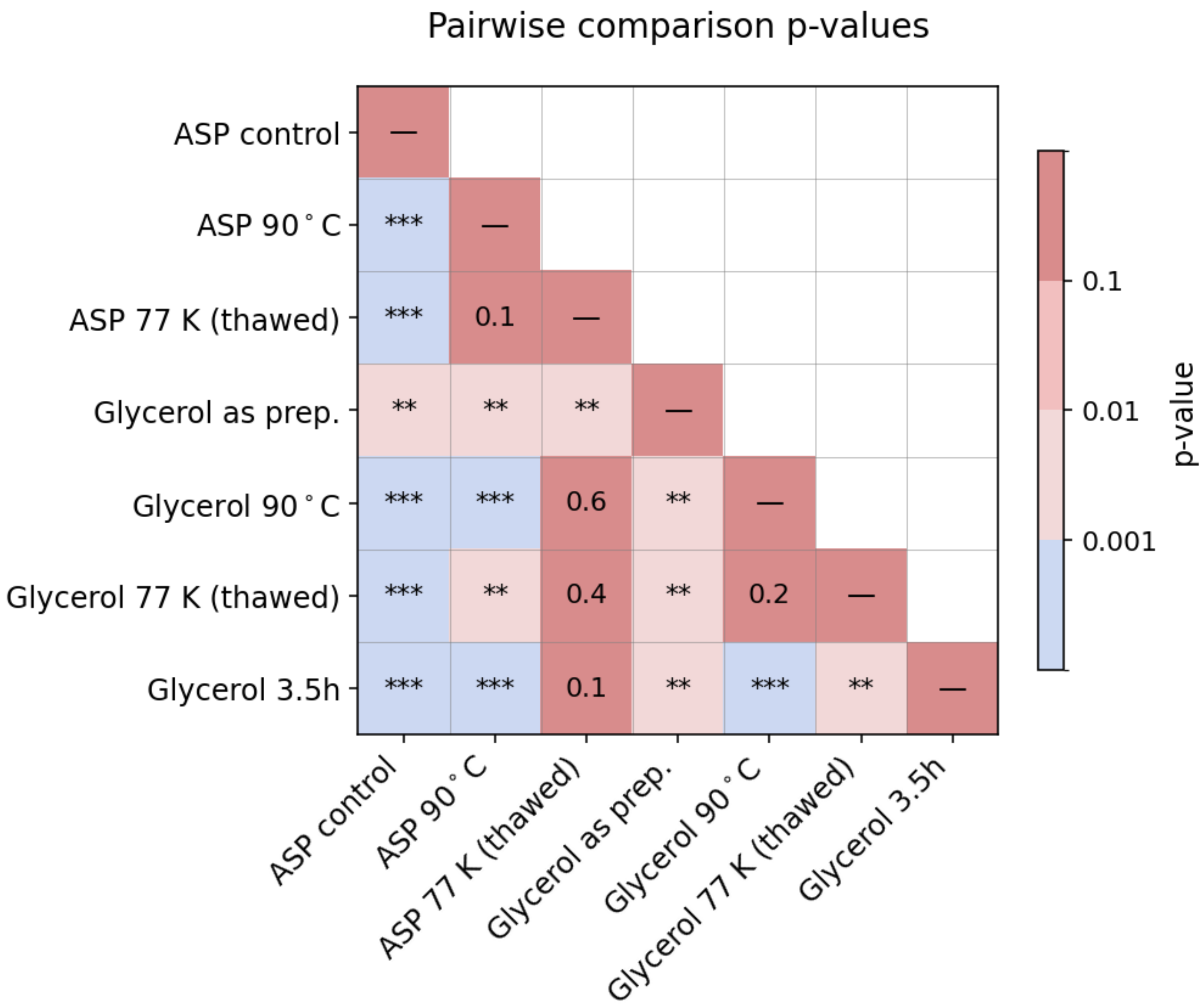

Figure S18. Pairwise comparison of the portion of dead cells between all the groups measured. Pairwise differences considered as significant if $p < 0.05$ Refers to **Figure 1F**.

Table S6. Pairwise comparison of τ_{AVG} between the cells which were transferred to ASP:glycerol medium (2:3) and Control cells (in ASP) – time- and concentration dependence data, correspond to **Figure 2 (A, B)** and **Figure 2 (C, D)**, respectively.

**λ_{em}, nm**	**Time**	***p*-value**	**Concentration, %**	***p*-value**
**685 nm**	0 min	0.001	10	0.016
	30 min	0.042	20	0.044
	60 min	<0.001	30	0.038
	90 min	<0.001	40	0.003
	120 min	0.001	50	0.016
	3.5 h	0.004	60	0.048
**720 nm**	0 min	<0.001	10	0.025
	30 min	0.075	20	0.045
	60 min	0.033	30	0.048
	90 min	0.005	40	0.044
	120 min	<0.001	50	0.045
	3.5 h	0.008	60	0.015

Statistical method applied was unpaired two-tailed Welch's t-test (unequal variances assumed).
Data shown in the Table: *p*-values for each comparison.
Sample size was n=3.
Groups with p <0.05 were considered statistically significant.
*** <0.001, ** <0.01, * <0.05

Table S7. Pairwise comparison of τ_{AVG} of cells between Control, DCMU- and FCCP-treated cells, measured at 685 nm and 720 nm emissions, across all temperatures (Refers to **Figure 6**):

**λ_{em}, nm**	**Temperature**	**Control vs. DCMU (*)**	**Control vs. FCCP (#)**	**DCMU vs. FCCP (&)**
**685 nm**	16 K	0.041	0.513	0.023
	40 K	0.024	0.579	<0.001
	77 K	0.674	0.455	0.563
	100 K	0.064	0.243	0.432
	130 K	0.011	0.141	0.416
	180 K	0.002	0.134	0.710
	230 K	0.882	0.135	0.128
	270 K	0.002	0.013	0.001
	RT	<0.001	0.661	<0.001
	RT (thawed)	0.520	0.085	0.099
	RT (thawed, 24 h)	0.053	0.102	0.268
**720 nm**	16 K	0.002	<0.001	0.154
	40 K	0.014	0.015	0.906
	77 K	0.001	0.030	0.863
	100 K	0.014	0.102	0.750
	130 K	0.024	0.209	0.652
	180 K	0.039	0.156	0.865
	230 K	0.274	0.109	0.136
	270 K	0.028	0.004	<0.001
	RT	0.001	0.509	0.001
	RT (thawed)	0.090	0.469	0.131
	RT (thawed, 24 h)	0.069	0.373	0.339

Statistical method applied was unpaired two-tailed Welch's t-test (unequal variances assumed).
Data shown in the Table: *p*-values for each comparison.
Sample sizes was as follows: Control n=5, DCMU n=5, FCCP n=3.
Groups with $p < 0.05$ were considered statistically significant.
*** <0.001, ** <0.01, * <0.05

Table S8. Pair-wise comparison of τ_{AVG} of cells after thawing to the respective before-freezing (BF) value (untreated and FCCP separately) and comparing FCCP to Control at the same time point after thawing (AT). (Refers to **Figure 8**):

λ_{em}, nm	t (AT), min	Untreated vs. BF (*)	FCCP vs. BF (#)	FCCP (AT) vs. Untreated (AT) (&)
685 nm	0	<0.001	0.007	0.046
	15	0.001	0.013	0.042
	30	0.001	0.031	0.290
	45	0.003	0.570	0.208
	60	0.003	0.375	0.014
	75	0.014	0.329	0.162
	90	0.005	0.351	0.046
	105	0.008	0.336	0.036
	120	0.010	0.214	0.032
	135	0.006	0.218	0.030
	150	0.009	0.184	0.011
	205	0.006	0.213	0.010
720 nm	0	<0.001	0.151	0.381
	15	<0.001	0.167	0.391
	30	0.001	0.225	0.822
	45	<0.001	0.692	0.022
	60	0.001	0.293	0.004
	75	<0.001	0.262	0.026
	90	0.001	0.343	0.012
	105	0.003	0.356	0.036
	120	0.001	0.307	0.043
	135	0.001	0.262	0.006
	150	0.001	0.260	0.049
	205	0.001	0.259	0.031

Statistical method applied was unpaired two-tailed Welch's t-test (unequal variances assumed).
Data shown in the Table: *p*-values for each comparison.
Sample sizes was as follows: Control n=3, FCCP n=3.
Groups with p <0.05 were considered statistically significant.
*** <0.001, ** <0.01, * <0.05 for Untreated vs. Untreated BF
<0.001, ## <0.01, # <0.05 for FCCP-treated vs. FCCP-treated vs. BF
&&& <0.001, && <0.01, & <0.05 for FCCP-treated (AT) vs. Untreated (AT)
